# Deciphering the complex three-way interaction between the non-integrin laminin receptor, galectin-3 and *Neisseria meningitidis*

**DOI:** 10.1098/rsob.140053

**Published:** 2014-10-01

**Authors:** Fulwah Alqahtani, Jafar Mahdavi, Lee M. Wheldon, Matthew Vassey, Necmettin Pirinccioglu, Pierre-Joseph Royer, Suzan M. Qarani, Shaun Morroll, Jeroen Stoof, Nicholas D. Holliday, Siew Y. Teo, Neil J. Oldfield, Karl G. Wooldridge, Dlawer A. A. Ala'Aldeen

**Affiliations:** 1 School of Life Sciences, Centre for Biomolecular Sciences, University of Nottingham, University Park, Nottingham NG7 2RD, UK; 2 Department of Chemistry, University of Dicle, 21280 Diyarbakir, Turkey

**Keywords:** LAMR1, RPSA, galectin-3, 37LRP, 67LR, *Neisseria meningitidis*

## Abstract

The non-integrin laminin receptor (LAMR1/RPSA) and galectin-3 (Gal-3) are multi-functional host molecules with roles in diverse pathological processes, particularly of infectious or oncogenic origins. Using bimolecular fluorescence complementation and confocal imaging, we demonstrate that the two proteins homo- and heterodimerize, and that each isotype forms a distinct cell surface population. We present evidence that the 37 kDa form of LAMR1 (37LRP) is the precursor of the previously described 67 kDa laminin receptor (67LR), whereas the heterodimer represents an entity that is distinct from this molecule. Site-directed mutagenesis confirmed that the single cysteine (C^173^) of Gal-3 or lysine (K^166^) of LAMR1 are critical for heterodimerization. Recombinant Gal-3, expressed in normally Gal-3-deficient N2a cells, dimerized with endogenous LAMR1 and led to a significantly increased number of internalized bacteria (*Neisseria meningitidis*), confirming the role of Gal-3 in bacterial invasion. Contact-dependent cross-linking determined that, in common with LAMR1, Gal-3 binds the meningococcal secretin PilQ, in addition to the major pilin PilE. This study adds significant new mechanistic insights into the bacterial–host cell interaction by clarifying the nature, role and bacterial ligands of LAMR1 and Gal-3 isotypes during colonization.

## 2. Introduction

The non-integrin laminin receptor (LAMR1), also commonly referred to as ribosomal protein SA (RPSA), is a highly conserved multi-functional protein that has been localized to the cell surface, the cytosol, the 40S ribosomal subunit and, in histone, chromatin and membrane-associated complexes in the nucleus [1–6]. We recently demonstrated that the three major aetiological agents of bacterial meningitis, *Neisseria meningitidis, Streptococcus pneumoniae* and *Haemophilus influenzae*, engage LAMR1 on the surface of human cells via specific surface ligands [7]. LAMR1 has also been identified as the surface receptor for the *Escherichia coli* K1 toxin Cfr1, a number of viruses, and the cellular prion protein [8–13]. LAMR1 also has roles in cell viability, adhesion and motility. Importantly, elevated LAMR1 expression correlates strongly with increased invasiveness and metastatic potential of cancer cells [2,5,14–16]. Taken together, LAMR1 has important functions in diverse pathological processes, particularly of infectious or oncogenic origins.

LAMR1 is encoded by the *RPSA* gene, which is present in 64 copies, although these are predominantly pseudo-genes; it is unclear how many functional copies there are [17], but one or more active genes encodes the 37 kDa ‘precursor’ protein (37LRP), which migrates at 37–45 kDa on SDS–PAGE gels. A proportion of 37LRP migrates to the cell surface, where it is thought to ‘mature’, possibly via dimerization and/or posttranslational modification, into the 67 kDa high-affinity receptor (67LR), which migrates at 60–67 kDa on SDS–PAGE gels. The transition from 37LRP to 67LR has never been directly demonstrated, however. It is the 67LR isoform that is thought to act as the receptor for laminin and/or other substrates including elastin or collagen [2,18–21], although the 37LRP isoform has also been suggested to have laminin-binding properties [18,22]. A 120 kDa form of the protein has also been described, but its nature, identity and relationship to 37LRP is unknown [23,24].

37LRP has also been suggested to associate with the β-galactoside-binding lectin galectin-3 (Gal-3), previously known as the 31 kDa human laminin-binding protein (HLBP31), to form a composite receptor for molecules including laminin [23]. Indeed, a heterodimer containing 37LRP and a protein carrying one or more galectin-3 epitopes has been proposed as the basis for 67LR [23]; this has given rise to confusion in the literature between the homo- and heterodimers. Evidence for heterodimerization has so far been circumstantial, based primarily on (i) the cross-reactivity of anti-Gal-3 antibodies with a 67 kDa protein [23], (ii) that Gal-3 and a 67 kDa protein can be co-eluted from laminin affinity columns by lactose, galactose and *N*-acetyl-lactosamine [25], and (iii) pre-treatment of laminin with β-galactosidase abolishes the interaction of the 67 kDa molecule and Gal-3 with laminin [25]. Nevertheless, 67LR is observed in Neuro 2a (N2a) cells, which do not express Gal-3, suggesting that 67LR and 37LRP–Gal-3 heterodimer exist as distinct, but potentially coexisting cell surface populations.

Together with galectin-1, Gal-3 is an important mediator of inflammation, and is involved in recruitment of neutrophils, bacterial recognition and activation of the phagocytic respiratory burst [26]. Gal-3 is the only chimera-type galectin, and occurs mainly as a monomer in solution [27]; it also self-associates on surfaces and, upon binding to divalent ligands, into homodimers or pentamers [28–30]. Gal-3 has one C-terminal 135-amino acid carbohydrate recognition domain (CRD) that is responsible for binding the β-galactoside moiety of mono- or oligosaccharides on several host molecules, including the β-galactosides and the poly-*N-*acetyl-lactosamine residues of host laminin [31], and structurally similar microbial molecules including neisserial lipooligosaccharide (LOS) [32,33]. The N-terminal domain (NTD) of Gal-3 is non-lectin-binding, but can bind proteins as well as recognizing the lipid A/inner core region of bacterial lipopolysaccharides (LPSs) [34]. It is also thought to be responsible for the protein's self-association, although Gal-3 self-association in solution via its CRD has also been described [35].

A fundamental understanding of the nature and functions of Gal-3 and the multiple isotypes of LAMR1 is central to our understanding of the diverse pathological processes in which these proteins play critical roles. LAMR1 and Gal-3 are both differentially expressed in various cancer cells, and between them play regulatory roles in a broad range of processes, including cancer cell growth, transformation, apoptosis, angiogenesis, adhesion, invasion and metastasis [2,36]. The direct relationship between these proteins remains controversial, and is often confusing. As a consequence, our current lack of understanding of the basic biochemistry, cell biology and ligand-specificity of the homo- and heterodimers of 37LRP and Gal-3 remains a hindrance to further progress towards deciphering these important pathological processes.

In this study, we show evidence for the self- and mutual association of 37LRP and Gal-3, show their distinctive surface distribution and demonstrate that they target an overlapping repertoire of surface ligands on *N. meningitidis*. We provide the first evidence that, together with LAMR1, Gal-3 expression enhances bacterial host–cell invasion. Our data clarify much of the confusion relating to LAMR1/Gal-3 monomeric and dimeric isotypes, and will have significant implications in the fields of infection and cancer biology.

## 3. Results

### 3.1. Molecular modelling supports the proposed association of 37LRP and Gal-3

**Figure 1.**
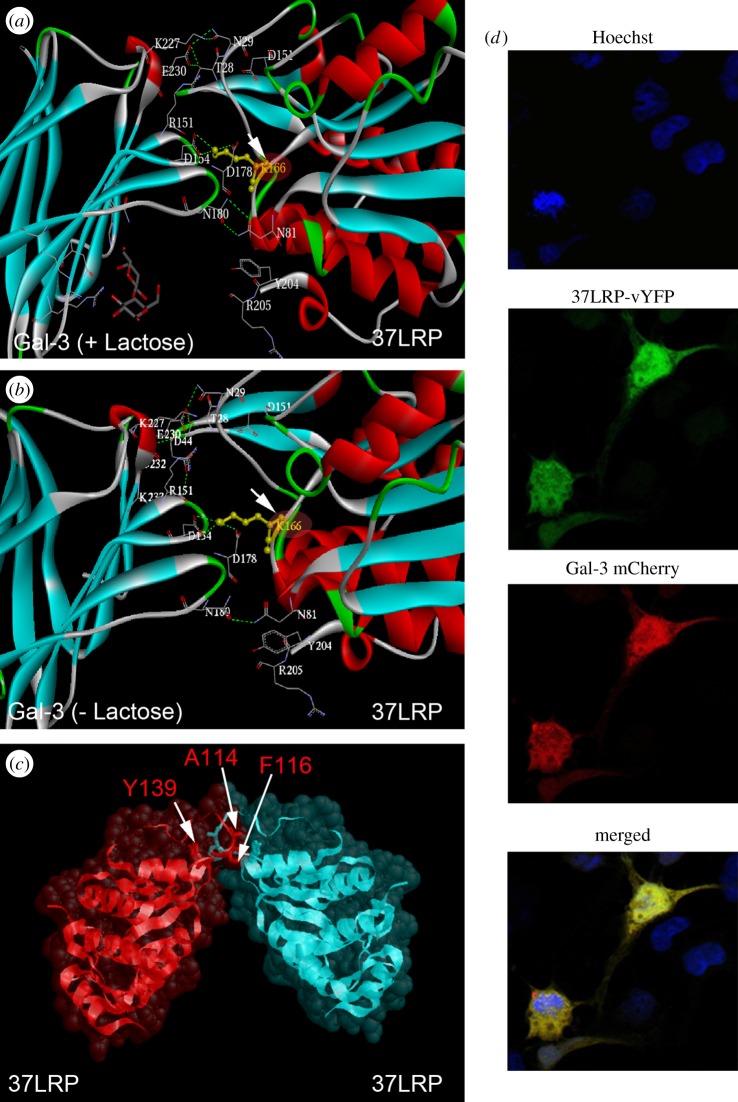
Molecular modelling of 37LRP interaction with galectin-3. (*a*) Ribbon diagram of the heterodimer interaction of 37LRP with (*a*) lactose-liganded or (*b*) non-lactose-liganded Gal-3. Residues involved in the hypothetical large protein–protein interface are represented with sticks. (*c*) Ribbon diagram with superimposed molecular surface of the homodimer interaction of 37LRP (red) with a second 37LRP (cyan) as proposed by Jamieson *et al.* [18]. (*d*) Fluorescently labelled recombinant Gal-3 and 37LRP were detected in transiently co-transfected COS7 cells via vYFP or mCherry tags. Cells were fixed at 24 h with 4% paraformaldehyde without permeabilization and stained with Hoechst.

The proposed interaction of 37LRP and Gal-3 was investigated using the ZDOCK server (zdock.umassmed.edu) and their docking compared with the previously proposed interaction of two 37LRP molecules forming a homodimer [18]. ZDOCK has been reported to achieve high predictive accuracy on protein–protein docking benchmarks for rigid-body cases and consistent success in the international protein–protein docking experiment [37]. The model revealed a large protein–protein interface between the 37LRP and Gal-3 subunits, involving several prominent 37LRP residues, mainly through salt bridges and hydrogen bonds (figure 1). In the proposed complex between 37LRP and lactose-associated Gal-3 (figure 1*a*), four salt bridges are apparent. They are K^166^ of 37LRP with D^178^ and D^154^ of Gal-3, R^80^ of 37LRP with D^178^ of Gal-3, D^151^ of 37LRP with R^151^ and K^227^ of Gal-3, and D^44^ of 37LRP with K^233^ of Gal-3. Hydrogen bonds between N^81^ of 37LRP and N^180^ of Gal-3, N^29^ of 37LRP and K^233^ and E^230^ of Gal-3, and Y^28^ of 37LPR and E^230^ of Gal-3 are also involved in the interaction (figure 1*a*). A similar interaction mode occurs between 37LRP and non-liganded Gal-3 (figure 1*b*). Here, K^166^ in 37LRP also interacts with D^178^ and D^154^ of Gal-3, and S^233^ of Gal-3 forms a hydrogen bond with D^44^ in 37LRP.

Importantly, binding of Gal-3 to the proposed heterodimer interface is incompatible with both homodimer formation (figure 1*c*) and laminin binding to at least one of the four proposed laminin-binding sites of 37LRP, because the relevant residues (A^114^, F^116^ and Y^139^ for homodimerization and F^32^, E^35^ and R^155^ for 37LRP-laminin binding) [38] are either sterically hindered or implicated in the heterodimer interaction. In short, our model supports a hypothesis of the mutual exclusivity of 37LRP homodimerization to form 67LR and 37LRP/Gal-3 heterodimerization for a given 37LRP molecule, while allowing for the possibility that both homo- and heterodimers could coexist as separate molecules. It should be noted, however, that the LAMR1 crystal structure is derived from a truncated protein and the proposed structure of the homodimer is highly speculative; it is possible that the proposed dimer interface may be an artefact of crystallization.

### 3.2. 37LRP and Gal-3 co-localization on the cell surface

To demonstrate cell surface co-localization of Gal-3 and 37LRP, two independent approaches were adopted. First, COS7 cells were co-transfected with constructs encoding recombinant 37LRP and Gal-3 proteins fused at their C-terminal ends with vYFP (YFP) or mCherry, respectively. Both proteins were successfully expressed and co-localized (figure 1*d*).

As the data presented in figure 1*d* could also be consistent with a cytosolic location of the protein, to demonstrate surface localization human cerebrovascular endothelial cells (hBMECs) were double-labelled for endogenous 37LRP, 67LR and/or Gal-3. Use of a wide range of antibodies with differing specificities for various isoforms of LAMR1 has resulted in considerable confusion in the literature. We selected antibodies with defined specificities as follows: rabbit IgG polyclonal (IHLR) raised in-house against 37LRP-derived peptide aa263–282 [7] and the commercially available mouse IgG_2b_ monoclonal (A7) raised against the 37LRP-derived peptide aa253–289 were employed to detect 37LRP. Both recognized an indistinguishable approximately 40 kDa protein in human hBMECs in immunoblotting experiments and produced interchangeable staining patterns in immunostaining experiments (electronic supplementary material, figure S1; co-localization coefficient: 81.5%). 37LRP was predominantly cytoplasmic but was also present to a much lesser extent in the membrane fraction of hBMECs and in the soluble nuclear fraction (electronic supplementary material, figure S1). IHLR, but not A7, exhibited very low level cross-reactivity with an approximately 60 kDa band (possibly 67LR) in the cytoskeleton-enriched cell fraction of hBMECs.

**Figure 2.**
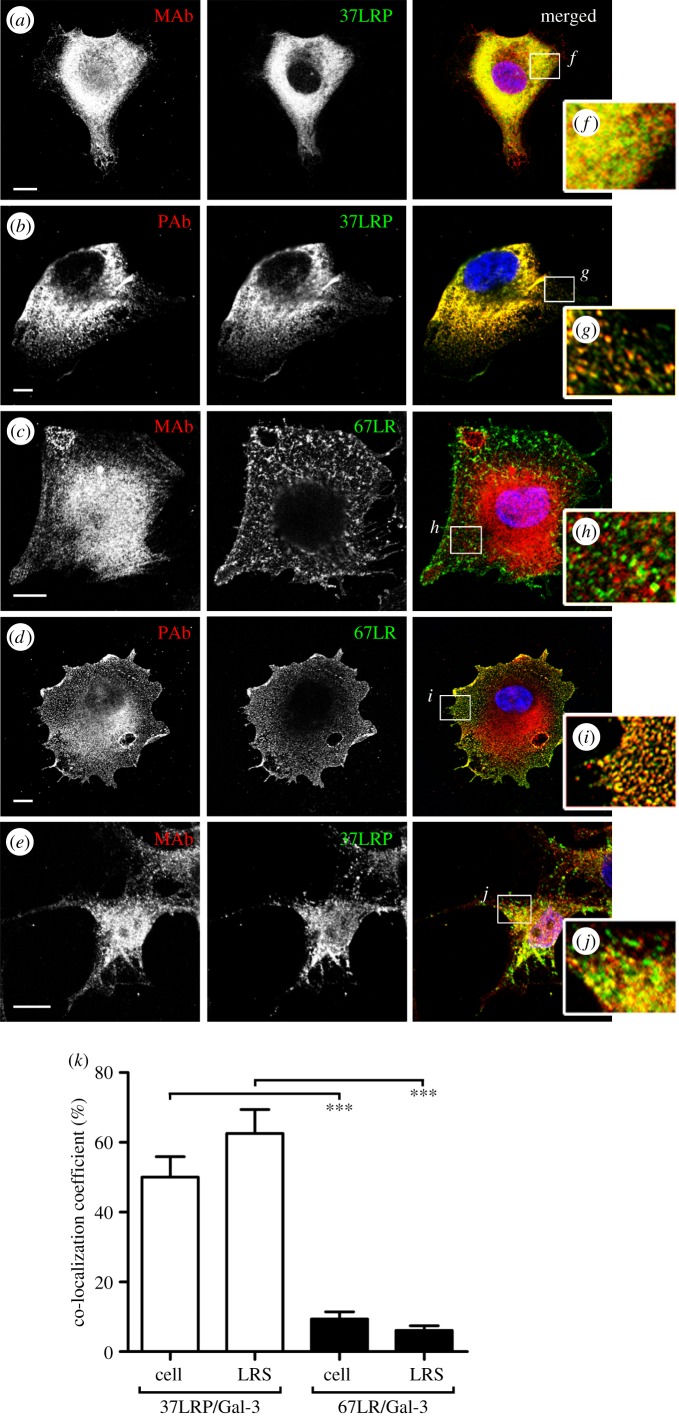
Preferential co-localization of galectin-3 and 37LRP. hBMECs were co-stained with either (*a*,*b*) 37LRP or (*c*,*d*) 67LR antibodies (green) in combination with (*a*,*c*) monoclonal (MAb M3/38) or (*b*,*d*) polyclonal (PAb) Gal-3 antibodies (red). (*e*) COS7 cells were stained with 37LRP and MAb Gal-3 antibodies. Co-localization is observed as yellow in merged images with Hoechst 33258 DNA staining (blue). Insets (*f–j*) are shown at 4× original magnification. Scale bar, 10 µm. Images are representative of ≥2 independent experiments. (*k*) Co-localization of LRP and 67LR with Gal-3 on the cell body of hBMECs was analysed by Zen software. Data are mean ± s.e.m., derived from >50 cells in ≥2 independent experiments. Data analysed by one-way ANOVA and Tukey test, ***p < 0.0001.

The widely used mouse IgM monoclonal MLuC5, which is known to block several LAMR1-mediated functions, exhibited a distinct immunostaining pattern from the two 37LRP-specific antibodies and predominantly recognized an approximately 60 kDa protein (presumably 67LR) in hBMEC cellular fractions. This protein was predominantly associated with the cytoskeletal cell fraction, but was also present in small amounts in the membrane and nuclear fractions (electronic supplementary material, figure S1). The commercially available Gal-3-specific mouse monoclonal (MAb; Mac-2 clone M3/38; Biolegends) and goat polyclonal (PAb; AF1154; R&D Systems) antibodies were also employed. Staining of non-permeabilized hBMEC cells clearly demonstrated the co-localization of Gal-3 (using MAb) with 37LRP (figure 2*a,b*), but not with 67LR on the cell surface (figure 2*c*). Similar results were obtained using a second Gal-3 MAb (clone 9H3.2; Millipore). Cells stained with anti-Gal-3 PAb and 67LR-specific MLuC5 consistently demonstrated a significant co-localization of Gal-3-specific epitope(s) with 67LR (more than 90%), consistent with the existence of a Gal-3-specific epitope associated with the 67LR molecule [23] (figure 2*d*). The identity of this epitope is unknown, but it is not localized to galectin-3 as two independent galectin-3-specific monoclonal antibodies do not detect a protein in association with 67LR. Additionally, COS7 cells (which exhibit no surface-localized 67LR (electronic supplementary material, figure S2) demonstrated clear surface co-localization of 37LRP and Gal-3 (figure 2*e*), suggesting that 37LRP/Gal-3 heterodimers are distinct from 67LR, a finding which is consistent with the observation that 67LR can be detected in N2a cells, which do not express Gal-3 [9]. By determining the co-localization coefficient of 37LRP or 67LR with Gal-3 (using MAb M3/38) on hBMECs, we observed that Gal-3 associated with approximately half of the surface-localized 37LRP (50 ± 5.9%); by contrast, only 9.4 ± 2.1% of the surface-localized 67LR co-localized with Gal-3 (figure 2*k*).

### 3.3. Gal-3 and 37LRP form homo- and heterodimers

**Figure 3.**
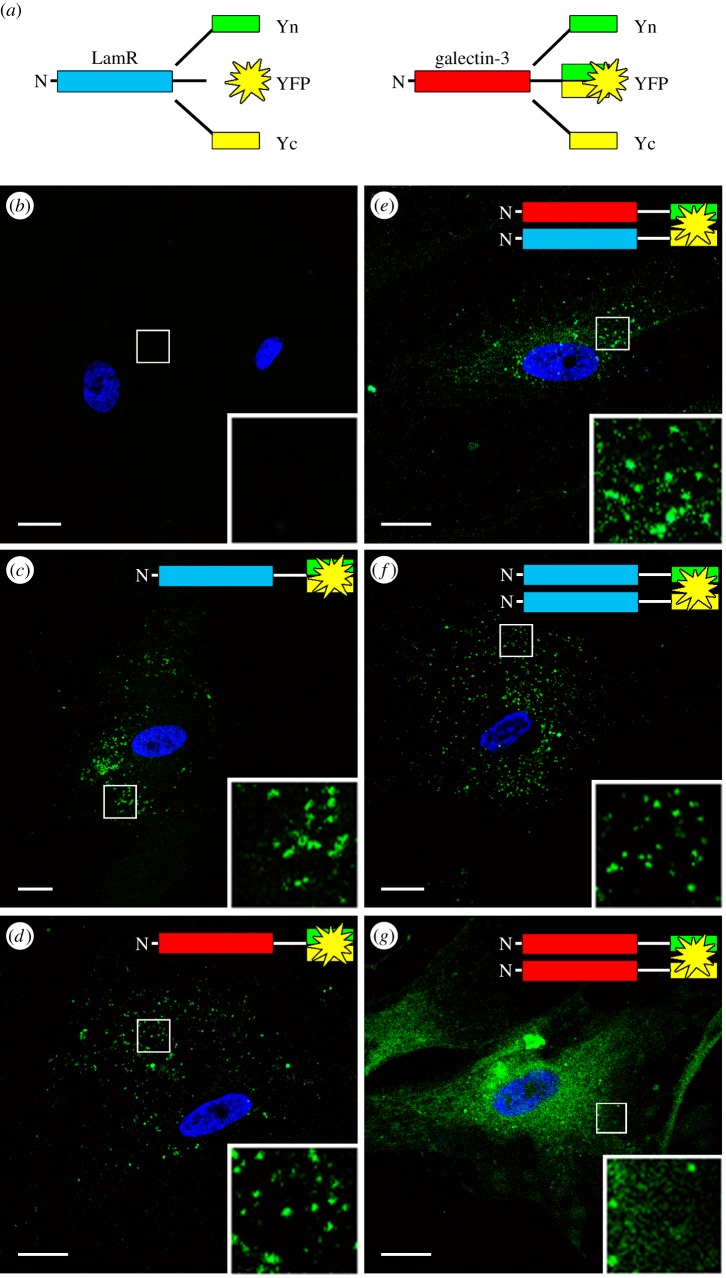
37LRP/Gal-3 hetero- and 37LRP/37LRP homodimerization is confirmed by BiFC analysis. (*a*) A schematic showing the different constructs used for transfection and subsequent BiFC analysis. LamR (magenta) or galectin-3 (red) cDNA was cloned in frame with Yn, YFP or Yc. Cells were then transfected as indicated (*b–g*) and allowed to express for 24 h prior to fixation and confocal analysis. Merged images also show Hoechst 33258 DNA staining. Scale bar, 10 mm. Insets are shown at 4× original magnification. Images are representative of ≥3 independent experiments.

To test the hypothesis that 37LRP is capable of forming homodimers and also heterodimers with Gal-3, as suggested by our model, we employed a bimolecular fluorescence complementation (BiFC) technique. A fluorescing union is formed when two separate non-fluorescent YFP subfragments are brought together by the close association of two intimately interacting proteins [39]. In addition to the full-length YFP, its N- or C-terminal domains (Yn and Yc, respectively) were fused to the C-terminal end of Gal-3 and 37LRP (figure 3*a*), and transfected into N2a, hBMECs and COS7 cells (figure 3*b–g* shows N2a cells). Immunoblot analysis of host cells (electronic supplementary material, figure S3) confirmed transfection and protein expression. Although 37LRP was readily observed in COS7 cells by fluorescence microscopy (electronic supplementary material, figure S2) and immunoblotting (electronic supplementary material, figure S3), no 67LR was observed in these cells, suggesting that under the experimental conditions tested, 67LR did not form. As the tagged proteins do not form a higher molecular mass protein equivalent to 67LR, it is possible that additional factors may be required subsequent to homodimerization (as detected by our BiFC experiments) for the maturation into the SDS-stable 67LR form of the protein, which are not present in these cells. This would be consistent with the observation that endogenous 37LRP was apparent in these cells (electronic supplementary material, figures S2 and S3), whereas 67LR was not detected. It should also be noted that in cells expressing endogenous 37LRP and 67LR the latter species runs in SDS–PAGE gels with an apparent molecular mass of around 60 kDa, which is lower than might be expected of a homodimer of 37LRP, which has an apparent molecular mass of around 42 kDa (electronic supplementary material, figure S1). The most likely explanation for this is that 67LR is resistant to denaturation in SDS, and thus maintains a more compact structure than during SDS–PAGE and runs with a higher electrophoretic mobility.

Cells were examined 24 h post-transfection by confocal microscopy after fixation without permeabilization. Transfection with full-length YFP-fused proteins (figure 3*c,d*, respectively; positive control) resulted in the appearance of punctate fluorescence; non-transfected cells (figure 3*b*) exhibited negligible fluorescence, as did cells transfected with Yn or Yc constructs alone (figure 5*d* shows FACS analysis of COS7 cells; see below). Co-transfection of cells with 37LRP–Gal-3, 37LRP-37LRP or Gal-3-Gal-3 (each pair carrying complementary Yn + Yc) yielded fluorescent cells, indicating both homo- and heterodimerization of both molecules (figure 3*e–g*). Similar results were observed in COS7 cells, whereas in hBMEC cells, punctate fluorescence could be observed in cells co-transfected with 37LRP–Gal-3 or 37LRP-37LRP, while cells co-transfected with Gal-3–Gal-3 did not fluoresce. The reason for this is not known. Quantification and statistical analysis of surface fluorescence of transfected cells, using confocal and FACS analysis, confirmed the statistical significance of homo- and heterodimerization of both 37LRP and Gal-3 (figure 5*d* shows data for COS7 cells).

### 3.4. Surface 67LR is resilient to siRNA knockdown of Gal-3 or 37LRP

**Figure 4.**
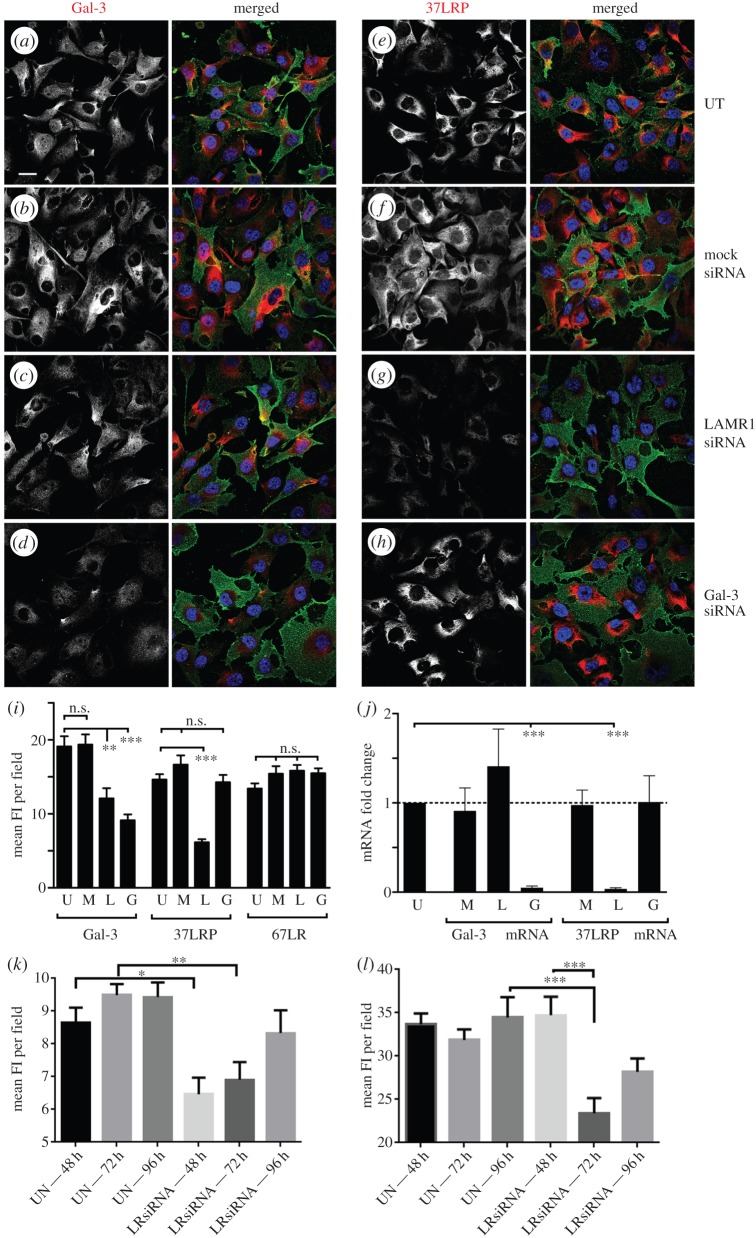
67LR is resilient to siRNA treatment of either Gal-3 or LAMR1. Early passage hBMECs were (*a*,*e*) left untreated or treated with (*b*,*f*) mock siRNA, (*c–g*) *RPSA* (LAMR1) siRNA or (*d–h*) *LGALS3* (Gal-3) siRNA. Following 48 h incubation, cells were fixed and co-stained for either (*a–d*) Gal-3 or (*e–h*) 37LRP (red) and 67LR. Left-hand panels show either Gal-3 or 37LRP (red) and merged images include 67LR (green) and DAPI DNA staining (blue). Scale bar (for all fields) in (*a*) = 25 mm. Images are representative of ≥3 independent experiments. (*i*) The mean fluorescence intensity (FI) of 37LRP, 67LR and Gal-3 was measured in each experimental condition. U, untreated; M, control siRNA; L, LAMR1 siRNA; G, Gal-3 siRNA. Data are mean ± s.e.m., derived from >100 cells in ≥2 independent experiments. Data analysed by one-way ANOVA and Dunnett test. n.s., not significant; **p < 0.01; ***p < 0.0001. (*j*) RT-qPCR analysis of Gal-3 and LAMR1 mRNA levels following mock (M), LAMR1 (L) and Gal-3 (G) siRNA treatment. Data are adjusted for GAPDH levels and normalized (fold change) to untreated cells (U; dotted line). In extended time frame, knockdown experiments cells were treated with non-targeting siRNA or RPSAsiRNA for 48, 72 or 96 h before fixing with 4% paraformaldehyde and stained with (*k*) anti-37LRP (A7; 1 : 1000) or (*l*) anti-67LR (MLuC5; 1 : 1000) followed by secondary Alexa antibody (Alexa 488 and 647). Images were acquired using a Zeiss LSM700. Bars represent mean fluorescence intensity (arbitrary units) + s.e.m. from at least 10 fields (minimum 30 cells field^−1^) measured across two independent experiments. Significance was determined by one-way ANOVA and Dunnett test. ***p < 0.0001; **p < 0.0002; *p < 0.05.

Gal-3 or 37LRP was knocked down by targeting *LGALS3* or *RPSA*, respectively, in hBMEC cells over a 48 h period (figure 4). RT-qPCR analysis confirmed the effective and specific silencing of Gal-3 and 37LRP siRNA treatment at the level of mRNA (figure 4*j*). Fixed siRNA-treated cells were co-stained for either Gal-3 or 37LRP and compared with untreated or mock siRNA-treated cells. *RPSA* siRNA-treated cells exhibited significantly reduced levels of both surface 37LRP and Gal-3 (figure 4*c,g*), whereas Gal-3 siRNA-treated cells exhibited significantly reduced surface levels of Gal-3 (figure 4*d*), but not 37LRP (figure 4*i*). Approximately 50% of surface-localized 37LRP was Gal-3-associated, and levels of surface-localized Gal-3 were significantly reduced (by approx. 37%) in the presence of 37LRP siRNA (figure 4*i*), implying that 37LRP promotes Gal-3 surface localization. It should be noted, however, that 37LRP is a ribosomal protein and it is also possible that depressed levels of this protein could reduce transcription of Gal-3. Surprisingly, in both 37LRP and Gal-3 siRNA-treated cells, the mean fluorescence intensity of 67LR was not significantly different from untreated (figure 4*a,e*) or mock siRNA-treated cells (figure 4*b,f*). Furthermore, overexpression of 37LRP by transfection did not coincide with increased 67LR (electronic supplementary material, figure S2). These observations indicate a low turnover of 67LR. To further investigate the relationship between expression of RPSA and the appearance of 37LRP and 67LR cells were treated with non-targeting siRNA or *RPSA* siRNA for 48, 72 or 96 h before fixing and analysis by confocal microscopy. 37LRP was significantly reduced by 48 h, as previously observed, and was still significantly reduced by 72 h, although by this time levels had started to recover. By 96 h, 37LRP levels had recovered further and were not significantly lower than in untreated cells (figure 4*k*). 67LR levels were not significantly different than in untreated or mock-treated cells by 48 h, as had previously been observed. However, by 72 h, there was an approximately 70% reduction in 67LR levels in *RPSA* siRNA-treated cells compared with untreated or mock-treated cells, which was highly significant. By 96 h, 67LR levels were still significantly depressed in *RPSA* siRNA-treated cells, although levels had started to recover by this time.

### 3.5. Gal-3 residue C^173^ and 37LRP residue K^166^ are implicated in 37LRP–Gal-3 dimerization

**Figure 5.**
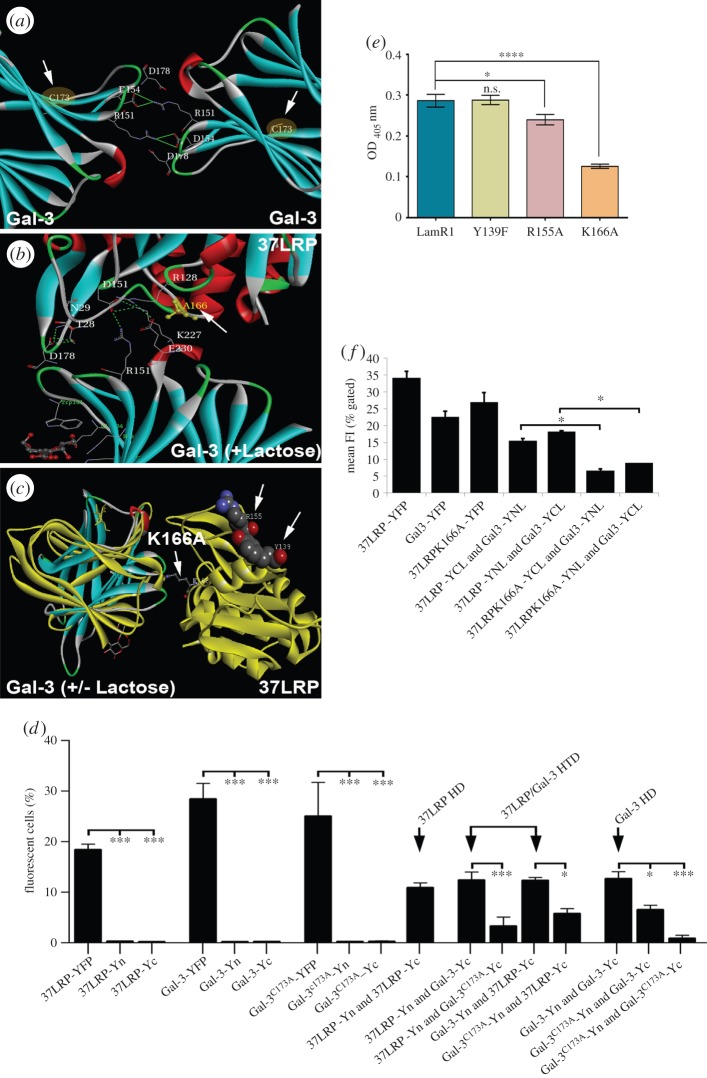
Gal-3 residue C^173^ and 37LRP residue K^166^ are implicated in 37LRP–Gal-3 dimerization. (*a*) Ribbon diagram of the homodimer interaction of Gal-3 with a second Gal-3 showing the position of C^173^ (white arrows). (*b*) Ribbon diagram with superimposed molecular surface of the heterodimer interaction of Gal-3 with 37LRP with residue 166 substituted to alanine (white arrow). The mutation of K^166^ in 37LRP to alanine is predicted to disrupt the interaction between the two molecules (compare with figure 1*a*), whereas some (i.e. between R^151^ and K^227^ of 37LRP with D^151^ of Gal-3) are retained. (*c*) Superimposed structures of lactose-liganded (yellow) and non-lactose-liganded (blue) Gal-3 complexed with 37LRP highlighting the positions of K^166^A, R^155^ and Y^139^ (white arrows). Other features in the 37LRP/Gal-3 interface are not shown for clarity. (*d*) Following transfection of COS7 cells with the indicated 37LRP and Gal-3 -vYFP, -Yn or -Yc fusion proteins, cells were harvested and the number of fluorescent cells quantified by FACS and expressed as a percentage of the total number of cells counted. Expression of 37LRP-YFP, Gal-3-YFP and Gal-3^C173A^-YFP resulted in a significant increase in fluorescent cells compared with -Yn and -Yc control constructs (37LRP: 18.4 ± 1.1% compared with 0.28 ± 0.07% and 0.18 ± 0.05%; Gal-3: 28.4 ± 3.1% compared with 0.19 ± 0.04% and 0.21 ± 0.03%; Gal-3^C173A^: 25 ± 6.7% compared with 0.21 ± 0.07% and 0.26 ± 0.12%). Co-transfection of 37LRP-Yn and -Yc, which would form a 37LRP homodimer (37LRP HD), resulted in 10.89 ± 0.9% fluorescent cells, a similar number to cells co-transfected with either 37LRP-Yn and Gal-3-Yc (12.39 ± 1.6%) or Gal-3-Yn and Gal-3-Yc (12.65 ± 1.4%), which would form a heterodimer (37LRP/Gal-3 HTD) or self-associated Gal-3 (Gal-3 HD), respectively. Similar results were obtained when Gal-3-Yn and 37LRP-Yc constructs were investigated. Mutation of C^173^ on Gal-3 resulted in a significant inhibitory effect on 37LRP/Gal-3 HTD (3.29 ± 1.8%; approx. 73% inhibition) and Gal-3 HD formation (6.5 ± 0.9%; approx. 49% inhibition). Co-transfection of Gal-3^C173A^–Yn and Gal-3^C173A^–Yc resulted in negligible numbers of fluorescent cells (0.8 ± 0.6%) demonstrating complete disruption of protein–protein interactions under these conditions. Data are mean ± s.e.m. of ≥3 independent experiments. (*e*) Binding of Gal-3 to ELISA wells coated with recombinant 37LRP or its mutant derivatives. The mean value obtained from Gal-3 binding to BSA-coated wells was subtracted from all data points. (*f*) Flow cytometry analysis of COS7 cells transfected with indicated pairs of BiFC constructs and analysed 36 h post-transfection. Substitution of LAMR1 lysine 166 with alanine significantly reduced its heterodimerization with Gal-3. Fluorescence intensity was evaluated as a percentage of gated cells. Data analysed by one-way ANOVA and Tukey test. ****p < 0.0001; *p < 0.05. Error bars = mean of triplicate values on three occasions ± s.e.m.

The sole cysteine residue of murine Gal-3 (C^186^, corresponding to C^173^ in its human homologue) has been implicated in Gal-3/Gal-3 homodimerization [40] (figure 5*a*). We substituted alanine for C^173^ (yielding Gal-3^C173A^), and fused the mutant molecule to Yn and Yc. Co-transfection of COS7 cells with complementary Gal-3^C173A^ Yn and Yc pairs resulted in very little fluorescence, confirming the central role of C^173^ in homodimerization and the validity of the BiFC approach (figure 5*d*). Dimerization of Gal-3^C173A^ with wild-type Gal-3 or wild-type 37LRP was also significantly reduced (figure 5*d*); the latter observation suggests a role for C^173^ in Gal-3/37LRP heterodimerization. In contrast, alanine substitution of 37LRP's two cysteines (C^148^ and C^163^) had no statistically significant effect on 37LRP cell surface expression cells as measured by FACS analysis of COS7 cells transfected with the relevant BiFC constructs (electronic supplementary material, figure S4).

Our molecular model suggested that K^166^ of 37LRP may play an important role in the interaction of this molecule with Gal-3 (figure 1). Substitution of alanine for K^166^ in 37LRP is predicted to disrupt several interactions between the two molecules (compare figure 1*a* and figure 5*b*; for clarity, the position of 37LRP K^166^ is highlighted in the proposed interaction of 37LRP with superimposed lactose and non-lactose-liganded Gal-3 shown in figure 5*c*). To investigate this, we purified recombinant 37LRP and a 37LRP^K166A^ derivative, and determined their binding to Gal-3 in ELISA experiments. The ability of the 37LRP^K166A^ derivative to bind rGal-3 was significantly reduced, confirming the involvement of this residue in heterodimer formation and supporting the validity of our model of heterodimerization (figure 5*e*). By contrast, substitution with phenylalanine or alanine, respectively, of key residues implicated in homodimerization or laminin binding (Y^139^ and R^155^, respectively) [38] had no significant impact on Gal-3 binding (Y139F), or reduced binding significantly but to a much lower degree (R155A; figure 5*e*). The involvement of K166 of LRP in binding to Gal-3 was confirmed by BiFC complementation experiments; K166A derivatives of 37LRP fused to either the N- or C-terminal portions of YFP interacted to a significantly lower degree with their corresponding C- or N-terminal YFP-labelled Gal-3 partners compared with the wild-type molecules (figure 5*f*).

### 3.6. Gal-3 engages meningococci and potentiates cellular invasion

**Figure 6.**
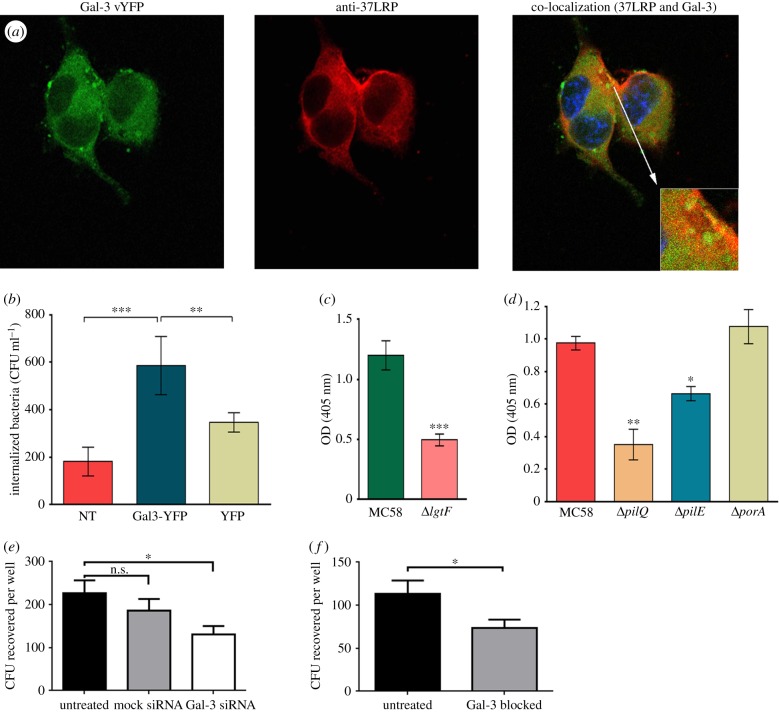
Gal-3 and 37LRP target common as well as specific meningococcal targets. (*a*) Subcellular localization of Gal-3.vYFP and endogenous 37LRP in transfected N2a cells. Cells were fixed at 24 h with 4% paraformaldehyde without permeabilization. Fluorescently labelled recombinant Gal-3 was detected via the vYFP tag. Endogenous 37LRP was detected using IHLR antibody. Co-localization is observed as yellow in merged image with Hoechst 33258 DNA staining (blue). Images are representative of ≥2 independent experiments. (*b*) Gal-3 expression enhances MC58 invasion of N2a cells. N2a cells were transiently transfected with an empty pcDNA3.1 zeo-vYFP vector alone (YFP), or the same vector containing Gal-3 (Gal-3-YFP). Cells were infected with MC58 for 4 h at a multiplicity of infection of 300 before treatment with 100 µg ml^−1^ gentamycin. NT, non-transfected negative control. Mean ± s.e.m. of at least three separate experiments. Significance was determined by Student's *t*-test. **p < 0.01; ***p < 0.001. (*c*) Binding of DIG-labelled *N. meningitidis* MC58 and its isogenic *lgtF* mutant to immobilized recombinant Lac–Gal-3. BSA-coated wells (negative control) were included and their mean value subtracted from those of the test. Mean ± s.e.m. of at least three separate experiments. Significance was determined by Student's *t*-test. ***p < 0.001. (*d*) Binding of DIG-labelled *N. meningitidis* MC58 and its isogenic *pilQ*, *pilE* and *porA* mutants to immobilized Lac–Gal-3. BSA-coated wells (negative control) were included and their mean value subtracted from those of the test. Mean ± s.e.m. of at least three separate experiments. Significance was determined by Student's *t*-test. *p < 0.05; **p < 0.01. (*e*) hBMEC cells were grown to 60% confluence in 24-well plates were transfected with siRNA oligomers specific for Gal-3, with mock siRNA or left untreated. Post-transfection cells (48 h) were incubated with 1 × 10^7^ freshly prepared meningococcal cells (strain MC58) per well. Post-infection cells (4 h) were treated with gentamicin (100 µg ml^−1^) for 1 h to kill extracellular bacteria. After 1 h incubatin monolayers were disrupted and homogenized with saponin and internalized bacteria enumerated by plating onto chocolate agar. Compared with non-transfected cells both specific and non-specific siRNA-treated cells demonstrated reduced levels of bacterial invasion, but numbers internalized into Gal-3 siRNA-treated cells were significantly lower than for non-transfected cells (one-way ANOVA; *p* < 0.05), whereas mock-transfected cell numbers were not significantly lower. Bars represent mean of four independent experiments (*n* = 42) and error bars indicate s.e.m. (*f*) hBMEC monolayers as above were pre-incubated with either anti-Galectin 3 monoclonal antibody (25 µg ml^−1^) or left untreated for 1 h. Wells were then treated as described above and numbers of invading meningococci enumerated. Significantly reduced invasion was observed in cells treated with anti-Gal-3 antibodies (unpaired *t*-test; *p* < 0.05). Bars represent the mean of four independent experiments (*n* = 30) and error bars represent s.e.m.

Gal-3 has previously been shown to be expressed in splenic tissue from patients infected with *N. meningitidis*, but not that of healthy humans, and co-localization of Gal-3 with meningococcal colonies on splenic tissue was demonstrated [33]. Quattroni *et al.* also showed that Gal-3 binds to the lactosamine moiety of meningococcal LOS. To determine whether interaction of meningococci with Gal-3 could potentiate cellular invasion, N2a cells, which do not express endogenous Gal-3, were transfected with a Gal-3-YFP construct prior to infection with meningococci. Diffuse expression of Gal-3 was confirmed by confocal microscopy (figure 6*a*). Expression of endogenous 37LRP, which co-localized with Gal-3.vYFP on the cell surface, was also confirmed (figure 6*a*), confirming that these cells were an appropriate model to study the role of Gal-3 in the interaction of meningococci with host cells. Internalized meningococci were measured using a gentamicin protection assay after infection of N2a cells transfected with either the empty vector pcDNA3.1 zeo-vYFP or the same vector containing the Gal-3-YFP construct. Meningococci invaded cells expressing Gal-3-YFP at significantly higher levels than those transfected with the empty vector, or non-transfected cells (figure 6*b*) confirming a role for Gal-3 in cellular invasion by meningococci. In order to confirm these findings in a more physiologically relevant cell line we knocked down Gal-3 expression by siRNA transfection in hBMEC cells and determined whether invasion of these cells by meningococci was affected. Indeed, transfection with Gal-3 siRNA significantly reduced invasion of these cells by meningococci (strain MC58), whereas transfection with a non-targeting siRNA had no significant effect (figure 6*e*). Similarly, blockading surface Gal-3 molecules by pre-treating hBMEC cells with polyclonal anti-Gal3 reduced the susceptibility of these cells to meningococcal invasion (figure 6*f*).

### 3.7. *Neisseria meningitidis* binds Gal-3 in a lipooligosaccharide-dependent and -independent manner

Quattroni *et al.* [33] showed that high concentrations (100 mM) of lactose significantly reduced, but did not abolish, binding of Gal-3 to meningococci. Similarly, meningococcal mutants not elaborating the α-chain of LOS showed significantly reduced, but not completely abolished Gal-3 binding (figure 6*c*). Collectively, this suggested the presence of additional meningococcal Gal-3 receptors. To explore this, we used lactose-liganded recombinant Gal-3 (Lac–Gal-3) in further ELISA assays. Lac–Gal-3 was biologically functional, as determined by its ability to bind 37LRP, *N. meningitidis* MC58 and 25 additional isolates, irrespective of their invasiveness, serotype or country of isolation (electronic supplementary material, figure S5 and table S1). Meningococcal binding to Lac–Gal-3 was not inhibited by increasing concentrations of lactose (up to 150 mM) or by adding a range of other Gal-3-binding sugars (Lewis X, Lewis Y, core H type II, galactose and fucose; electronic supplementary material, figure S6), suggesting that lactose-liganding had successfully blocked the carbohydrate-dependent (and thus LOS-dependent) binding capacity of Gal-3.

### 3.8. Gal-3 and 37LRP target common as well as specific meningococcal targets

To identify non-LOS meningococcal Gal-3-binding surface molecules, we employed a re-tagging (a contact-dependent cross-linking) approach. MC58 cells were incubated with Lac–Gal-3 conjugated to a light-activated cross-linker. Following photoactivation and subsequent exposure to reducing conditions to allow transfer of the reactive biotin moiety to molecules in close proximity to the Lac–Gal-3, bacteria were lysed and the biotin-tagged proteins purified with streptavidin-coated magnetic beads as previously described [41]. Extracted biotin-tagged proteins were separated by SDS–PAGE and identified using MALDI-TOF. This approach identified PilQ (accession: NMB1812; 82.4 kDa; score: 2463.12) and PilE (accession: P05431; 18.1 kDa; score: 2029.04) as the only Lac–Gal-3 binding meningococcal surface ligands. PilQ is a conserved outer membrane secretin that is absolutely required for type IV pilus extrusion [42]. PilE is the major glycosylated pilin subunit that forms the shaft of the hair-like and retractile type IV pilus fibre [43,44].

A role for each of these proteins in Lac–Gal-3 binding by whole bacteria was confirmed by testing the Lac–Gal-3 binding activity of *pilQ* and *pilE* mutants. As expected, MC58 mutants lacking *pilE* or *pilQ* showed significantly reduced Lac–Gal-3 binding when compared with wild-type (figure 6*d*). Mutation of PorA (a 37LRP-binding, surface-exposed, outer membrane protein employed as a negative control) had no significant effect on Lac–Gal-3 binding (figure 6*d*). Because PilQ is required for secretion and surface expression of the PilE-containing fibre, it is not possible from these data to distinguish between a direct role for PilQ binding of Gal-3 from an indirect effect owing to the lack of surface PilE. However, these data show that at least PilE expression is required for maximal Gal-3 binding.

## 4. Discussion

It is increasingly well established that 37LRP and Gal-3 have a complex functional relationship and are likely to play collaborative roles during physiological and pathological events, including the interaction of mammalian host cells with pathogens [2,45]. On the cell surface, each of these molecules has been suggested to be both self-associating as well as associating with each other. However, there is extensive confusion in the literature regarding the relationship between the 37LRP species, assumed to represent the monomer, and a 67 kDa ‘mature’ form of the protein, which has been described as either a homodimer or a heterodimer of 37LRP with Gal-3. Here, we present evidence for both self- and mutual association of both 37LRP and Gal-3. We employed molecular modelling to visualize the 37LRP–Gal-3 interaction supporting previous predictions of mutual association, and further informed the putative critical interfaces and key residues involved. BiFC experiments supported the hypothesis that 37LRP and Gal-3 both homo- and heterodimerize, and that the single cysteine (C^173^) of Gal-3 (but not the two cysteines of 37LRP) is critical for these associations, as was previously shown for the murine Gal-3 molecule's equivalent residue C^186^ [40]. We showed that monomeric 37LRP, its presumed homodimer 67LR and the 37LRP/Gal-3 heterodimer coexist as distinct populations on the host cell surface. It is of note that 67LR has previously been shown to incorporate a Gal-3-specific epitope that is not present on monomeric 37LRP [23,45]. It is likely that this epitope was responsible for the observed co-localization of 67-LR with one or more Gal-3 specific epitopes when a polyclonal antiserum was employed. We show that this is not due to the presence of Gal-3 in this complex, however, as it is not detected by two independent Gal-3-specific monoclonal antibodies. It is possible that homodimerization results in the creation of a new epitope that mimics a Gal-3 epitope. Alternatively, the polyclonal anti-Gal-3 antiserum may contain 67LR cross-reacting antibodies that are not specific for Gal-3. The latter possibility seems unlikely, however, as the polyclonal antiserum employed was raised against recombinant Gal-3 produced in *E. coli*. Gal-3 preferentially associated with 37LRP and rarely (if at all) with 67LR, and knockdown of Gal-3 had no discernable qualitative or significant quantitative effects on cell surface 67LR, confirming early models which suggested that Gal-3 is not a component of 67LR [9,23]. Interestingly, while 37LRP and Gal-3 are shown here to associate, these proteins dissociate under denaturing SDS–PAGE and thus cannot represent 67LR, which is known to be resistant to dissociation in the presence of SDS. Elevated cell surface 67LR is a prognostic marker for metastatic potential in cancer, yet overexpression of 37LRP by transfection is not coincident with increased 67LR, which is in line with previous observations [46–48]. Indeed, the transition of 37LRP to 67LR has not been conclusively demonstrated to date, and recombinant 37LRP has never been unambiguously shown to associate with higher molecular mass isoforms corresponding to the native 67LR isoform [47]. Our data support the hypothesis that 67LR is a homodimer of 37LRP, but it remains a possibility that the ability of 37LRP to homodimerize that we have demonstrated does not represent 67LR, and further experiments will be required to conclusively settle this matter. The delayed reduction in 67LR expression after prolonged siRNA knockdown of *RPSA* described here represents the clearest evidence yet that 67LR is indeed derived from the smaller protein. It should be born in mind, however, that another plausible explanation for the reduction in 67LR after prolonged knockdown of RPSA is due to a general reduction in protein synthesis that knockdown of 37LRP, a known ribosomal protein, results in a more generalized depression of new protein synthesis. While some 37LRP partitions to the membrane fraction, this precursor protein is predominantly isolated in soluble cytoplasmic cell fractions, where it is known to have a clearly defined ribosomal association and function. By contrast, 67LR has been clearly demonstrated previously to predominate in the membrane and, in particular, preferentially localizes to the environment of ‘lipid raft’ domains and focal plaques, co-localizing with vinculin and β-actinin [3,49]. Our demonstration that 37LRP and 67LR are essentially discrete populations implies that the majority of membrane-associated 37LRP is not lipid-raft-resident or focal-plaque-associated. While we acknowledge that transfection and high-level expression of recombinant proteins can give rise to anomalous protein localization, and that single amino acid changes can have an effect by altering the overall conformation of a protein, the observation that our data are consistent with the *in silico* modelling data supports our conclusions based on the BiFC experiments.

Therapeutic strategies based on 67LR currently involve selective drug targeting, antibodies against 37LRP, polysulfated glycans and siRNA treatment. Resolving the relationship and mechanism(s) governing ‘maturation’ of 67LR may provide new approaches in combinatorial cancer therapy. As interest in LAMR1 derives from its potential for therapeutic intervention, and the molecule is clearly a pathologically significant multi-functional protein involved in a wide variety of processes, assigning roles to differing receptor populations and determining any potential pleiotropism or functional redundancy will be critical for effective and selective targeting.

While the precursor–product relationship between 37LRP and 67LR is widely accepted, the mechanism of 67LR maturation remains unresolved. As we show that 37LRP is the predominant Gal-3-associated LAMR1 population, it is tempting to speculate that Gal-3 may play a regulatory or mechanistic role in this process. Indeed, modelling revealed that the interaction domains for 37LRP homo- and heterodimer formation may overlap, and thus form the basis for two mutually exclusive LAMR1 cell surface populations. In support of our hypothesis that a proportion of cell surface Gal-3 is spatially restricted to 37LRP, previous reports by Buto *et al.* [23] showed that treatment of A431 cells with cerulenin, to inhibit 37LRP acylation, resulted in a marked decrease in detectable Gal-3 and 67 kDa LAMR1 in the NP-40 soluble membrane fraction [23].

The fact that 37LRP and Gal-3 bind common and separate antigens in the human host further supports the hypothesis that the two proteins engage in a joint and mechanistically synchronized interaction during physiological and pathological events. Here, we show that this is paralleled during bacterial–host cell interactions. Using *N. meningitidis* as a model invasive organism that is known to interact with both Gal-3 and 37LRP [7,33], we showed that both host molecules are targeted by a common bacterial surface ligand (PilQ). The latter protein forms a large (960 kDa) homo-dodecamer complex with an apparent cavity through which the type IV pilus fibre is exported and retracted [50]. PilE is the major subunit that forms the shaft of that fibre [43]. PilQ and PilE remain intimately positioned and are surrounded by LOS, which is the predominant lipid moiety of the outer leaflet of the outer membrane. PorA, the other specific meningococcal ligand for 37LRP, is also a pore-forming outer membrane protein present in large numbers, and juxtapositioned with both PilQ and LOS [7,51]. Meningococci, and perhaps other bacterial pathogens, are likely to have evolved a closely positioned array of 37LRP- and Gal-3-binding surface ligands, with consequences for the virulence of these pathogens. Ligand–receptor interactions may act in concert to enhance cell–cell intimacy and affinity, and trigger downstream host cell-signalling and cytoskeletal rearrangements as a prelude to cellular invasion and tissue penetration [52].

**Figure 7.**
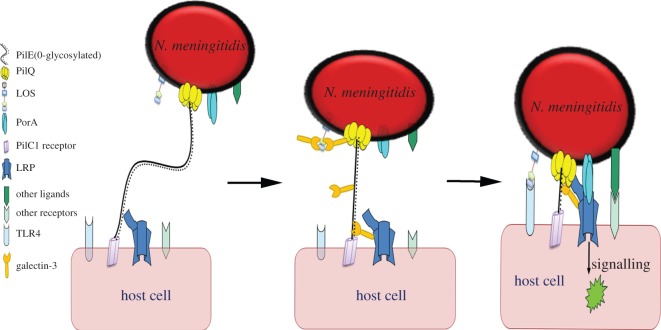
Illustration of meningococcal binding to host cells using surface ligands. Initial contact mediated by PilC1, which binds its putative receptor, followed by 37LRP/Gal-3-PilE binding, which strengthens the initial contact, and pilus retraction, which brings the two cells closer together. Decametric PilQ on the meningococci surface binds to both 37LRP and Gal-3, establishes intimate contact, strengthens interactions, and triggers host cell signalling events.

Taken together, these findings lead us to propose a model for the dynamic and mechanistic interaction of the host and bacterial surface proteins (figure 7). We propose that upon entry into the host (mucosa or blood stream), *N. meningitidis* would initiate contact with host cell surfaces via the type IV pili, which extend up to several micrometres, and at the tip may contain the adhesive PilC1 [44]. Retracting pili induces twitching motility that allows the organism to move over host cell surfaces against the tide of mucus or blood flow, until it comes into intimate contact with specific receptors [53]. Secreted forms of Gal-3 may bind LOS and/or bacterial protein ligands, a process that is likely to protect the host from bacterial colonization. However, meningococci may use Gal-3 to enhance its adherence to the host cell surface via Gal-3/37LRP heterodimerization, or shed the Gal-3–LOS complex as part of the outer membrane vesicles (which are well known to be shed continuously). Interestingly, surface Gal-3 is thought to be a negative regulator of the LOS-induced inflammatory response, and protects the host from endotoxin shock while also promoting survival of invading bacteria [54]. Similarly, when triggered by specific molecules (e.g. the LAMR1-binding green tea-derived polyphenol, EGCG), surface LAMR1 is believed to inhibit TLR4 and TLR2 responses in mouse macrophages [55,56].

Given that both 37LRP and Gal-3 are broadly distributed in different cellular compartments, their collaborative interaction with the meningococcus or its ligands may not be limited to the cell surface. In the cytoplasm, Gal-3 can bind to Bcl-2 and inhibit cell apoptosis, and in the nucleus it can regulate gene transcription [31,57]. Suppression of Gal-3 in highly malignant human breast carcinoma cells resulted in reversion of the transformed phenotype and led to the inhibition of tumour growth in immunologically suppressed mice [58]. Extracellular Gal-3 can bind to cell surface *N*-glycans and induce monocyte and T-cell apoptosis, which may help bacterial or tumour cell evasion from the immune surveillance. Gal-3 can suppress IL-5 production and inhibit B-lymphocyte differentiation, and increase phagocytosis of neutrophils. In human monocytes, Gal-3 is chemotactic and increases calcium influx at high concentrations, whereas at low concentrations it promotes chemokinesis [59].

In conclusion, we demonstrate the presence of discrete populations of LAMR1 on the epithelial and endothelial cell surface. We show that 37LRP can form homodimers (67LR) as well as heterodimers with Gal-3, which is also capable of forming homodimers. These surface molecules are likely to play distinct roles in a number of oncogenic, neurodegenerative and infectious diseases. 37LRP and Gal-3 target common and specific meningococcal surface ligands, events that are exploited by the organism to enhance its adhesion and cell invasion. Our data enhance our understanding of the roles of Gal-3 and 37LRP in host cell biology, which can be exploited for the design of therapeutic and/or preventive strategies against invasive bacterial and/or oncogenic diseases.

## 5. Material and methods

### 5.1. Cell culture

All cells were cultured at 37°C, 5% CO_2_. Human brain microvascular endothelial cells were cultured in endoprime media (PAA) supplemented with 5% (v/v) fetal bovine serum (FBS), IGF, bFGF, ascorbic acid, hydrocortisone and heparin, EGF, VEGF (all from PAA) and 1% antibiotic–antimycotic solution (v/v) (Gibco). Cells were either P2–15 primary cells (ScienCell, USA) or immortalized cells at either early (P10–30) or late (P60–70) passage. Where cells were routinely cultured on fibronectin, fibronectin-coated T75 flasks were used (BD Biosciences). Neuro 2a (N2a) mouse neuroblastoma cells were cultured in Eagle's minimum essential medium supplemented with 1% (v/v) antibiotic–antimycotic solution and 10% (v/v) FBS. COS7 cells were grown in Dulbecco's modified Eagle's medium (DMEM, Invitrogen) supplemented with 1% (v/v) antibiotic–antimycotic solution (Gibco) and 10% (v/v) FBS. Cell culture medium was changed every 2 days, and cells were split using trypsin–EDTA (Gibco) upon reaching 90% confluence.

### 5.2. Flow cytometry

COS7 cells were grown in six-well plates and detached 24 h after transfection using cell dissociation solution (Sigma), washed in PBA buffer (0.5% bovine serum albumin (BSA), 0.5% sodium azide in PBS), resuspended in 0.5% paraformaldehyde (PFA) in PBS (Oxoid) and subjected to flow cytometry using a Coulter Altra flow cytometer. Data acquisition and analysis were performed with Weasel v. 2.5 software. In each case, 50 000 cells were counted in triplicate and used to calculate the average BiFC signal intensity ± s.e. Full-length YFP fluorescence signal was used as the interassay reference for maximal fluorescence in each experiment against which the BiFC signal intensities were compared.

### 5.3. Immunofluorescence

For confocal analysis, cells were grown on acid-etched glass 12 mm coverslips (SLS) that were coated with 0.1% human fibronectin (1 µl 2.5 cm^−2^; Sigma) or laminin10 (1 µg cm^−2^; Millipore) as required. Following any treatments, cells were fixed with 4% PFA (10 min at RT) and, if required, permeabilized with 0.1% Triton X-100/1% BSA in PBS (5 min at RT). Following 1 h incubation in PBS/4% BSA to reduce non-specific binding, coverslips were incubated with primary antibody(s) either as a cocktail or sequentially, depending upon predetermined secondary antibody cross-talk: anti-67-kDa LAMR1 (MAb MLuC5, 1 : 100, Abcam); anti-37-kDa LAMR1 (PAb IHLR, 1 : 100 [7]; MAb A7, 1 : 250 (Santa Cruz Biotech.); anti-Gal-3 (MAb M3/38, 1 : 50 (Biolegends); MAb 9H3.2, 1 : 100 (Millipore); PAb, 1 : 50 (R&D Systems)) in PBS-T/4% BSA for 1 h. Coverslips were washed three times in PBS and incubated with conjugated secondary antibody(s): anti-mouse IgM (Alexa647, 1 : 1500; Alexa488, 1 : 1000); anti-mouse IgG (Alexa680, 1 : 200); anti-rabbit (Alexa488, 1 : 400; Alexa680 1 : 200); anti-goat (Alexa680, 1 : 200) [1 : 200]; anti-rat (Alexa647, 1 : 200) all from Molecular Probes, in PBS-T/4% BSA for 1 h and washed three times in PBS, once in H_2_O. Coverslips were mounted with Prolong Gold anti-fade with/without DAPI (Invitrogen) and images obtained as sections, unless stated otherwise, by confocal microscope. For each experiment, unstained and secondary antibody-alone samples were processed in parallel to control for non-specific staining. Additionally, all possible primary/secondary combinations were checked for cross-reactivity and sequential staining used as required. For all co-localization studies, samples stained for localization of each protein individually were processed in parallel and, in all cases, the fluorescence of adjacent channels was monitored for bleed through.

### 5.4. Confocal microscopy

Images (400–600 nm optical sections) were acquired using a Zeiss LSM 700 AxioObserver confocal microscope using a Plan-Apochromat 63×/1.40 Oil DIC M27 objective with Zen 2009 operating software. Images were processed using ImageJ and Adobe Photoshop software. Raw data are available upon request. Co-localization analyses used the Zen software function with an FI threshold of 50; the data were not intensity-weighted. Mean FI data of individual cells or fields of cells were also obtained using Zen software.

### 5.5. Bimolecular fluorescence complementation analysis

BiFC expression constructs were obtained by PCR: cDNA sequences of human LAMR1 (888 bp) and galectin-3 (725 bp) were generated with flanking N-terminal *Eco*RI/Kozak and C-terminal *Xho*I restriction sites using the following primer sequences: for galectin-3, 5′-GCGCGAATTCGCCATGGCAGACAATTTTTCGCT-3′ (forward primer) and 5′-GCGCCTCGAGTATCATGGTATATGAAGCACTGGTG-3′ (reverse primer); and for LAMR1, 5′-GCGCGAATTCGCCATGTCCGGAGCCCTTGAT-3′ (forward primer) and 5′-GCGCCTCGAGAGACAGTCAGTGGTTGCTCC-3′ (reverse primer). The amplified PCR products were digested and ligated into an *Eco*RI/*Xho*I-digested pcDNA3.1zeo vector, which contained either full-length Venus yellow fluorescent protein (vYFP) or the N- or C-terminal regions (Yn or Yc, respectively) of vYFP or full-length mCherry. Cysteine mutations were introduced by site-directed mutagenesis (QuikChange, Stratagene) using the following primer sequences: for Gal-3^C173A^, 5′-GAGAACAACAGGAGAGTCATTGTTGCCAATACAAAGCTGGATAATAACTG-3′ (forward primer) and 5′-CAGTTATTATCCAGCTTTGTATTGGCAACAATGACTCTCCTGTTGTTCTC-3′ (reverse primer); for 37LRP^C148A^, 5′-CCTACCTACCATTGCGCTGGCTAACACAGATTCTCCTCTG-3′ (forward primer) and 5′-CAGAGGAGAATCTGTGTTAGCCAGCGCAATGGTAGGTAGG-3′ (reverse primer); and for 37LRP^C163A^, 5′-TGGACATTGCCATCCCAGCCAACAACAAGGGAGCTC-3′ (forward primer) and 5′-GAGCTCCCTTGTTGTTGGCTGGGATGGCAATGTCCA-3′ (reverse primer). Cells were grown on fibronectin-coated 12 mm glass coverslips and transfected at approximately 60% confluence using TransIT-2020 at a DNA : TransIT-2020 ratio of 2 : 1 according to manufacturer's instructions. In brief, preformed DNA : TransIT complexes were added to the growth medium for 4 h. Cells were then washed with growth medium, and recombinant proteins expressed for 24 h before fixing with 4% PFA and subsequent confocal analysis.

### 5.6. siRNA of galectin-3 and LAMR1

hBMECs were cultured as described and transfected with predesigned siGENOME SMARTpool (mix of four siRNAs targeting one ORF) targeting LAMR1 (M-013303-01-0005), galectin-3 (M-010606-02-0005) or ON-TARGETplus non-targeting pool control siRNA (Dharmacon, Thermo Scientific). Cells were transfected on day 1 with a final concentration of 37.5 nM siRNA using the DharmaFECT transfection reagents (Dharmacon, USA). The inhibition of LAMR1 and Gal-3 expression was assessed at 48, 72 or 96 h post-transfection 3 by confocal analysis, quantitative polymerase chain reaction (RT-qPCR) and immunoblot analysis. To determine transfection efficiency, non-transfected and siGLO-transfected cells were analysed 1 day after transfection on a FACSCaliber machine (Beckton–Coulter). All galectin-3 or LAMR1 confocal images were acquired at the same resolution and scale, with the same hardware/laser settings used to image untreated control cells. Identical image manipulations were performed on all images stained with the same antibody set.

### 5.7. qPCR analysis

hBMECs were washed twice with serum-free EndoPrime base medium (PAA) and total RNA extracted using the RNeasy mini kit (Qiagen) according to the manufacturer's instructions. DNA was removed using RNAase-free turbo-DNAase I (Ambion, Applied Biosystem). RNA was cleaned and concentrated using RNeasy MiniElute clean-up kit (Qiagen). cDNA was synthesized using high-capacity cDNA reverse transcription kit (Applied Biosystems). RT-qPCR was performed in an ABI7500 real-time PCR system (Applied Biosystems) with the brilliant SYBR green qPCR master mix (Stratagene). Cycling was initiated at 95°C for 10 min, followed by 40 cycles of 95°C for 15 s, 60°C for 60 s and 60°C for 1 min. Samples were run in triplicate, and relative expression of LAMR1 and galectin-3 was calculated using the comparative threshold cycle method normalized to GAPDH. Primers were designed using Primer 3 software and obtained from Sigma-Aldrich. Primer sequences were as follows: GAPDH, 5′-GGGAAACTGTGGCGTGAT-3′ (forward primer) and 5′-TTCAGCTCAGGGATGACCTT-3′ (reverse primer); LAMR1, 5′-CCATTGAAAACCCTGCTGAT-3′ (forward primer) and 5′-CAGCGCAATGGTAGGTAGGT-3′ (reverse primer); and galectin-3, 5′-CTATAGCCGGGACTCCTTCC-3′ (forward primer) and 5′-AGTTCCAGGGCACATACGTC-3′ (reverse primer).

### 5.8. Purification of recombinant protein

Cells from 50 ml culture were re-suspended in 5 ml of buffer B (8 M urea (Sigma), 0.1 M NaH_2_PO_4_ (BDH) and 0.01 M Tris–HCl (Sigma) pH 8.0) and then sonicated in an ice bath for 15 cycles of 10 s with 5 s of cooling between cycles. Lysate was centrifuged at 10 000*g* for 30–40 min at 4°C to pellet cellular debris, followed by incubation of the supernatant with 20 mM imidazole (Qiagen) and cobalt resin (Fisher Scientific) overnight at 4°C. Supernatant was passed through a gravity column, extensively washed with buffer C (8 M urea, 0.1 M NaH_2_PO_4_ and 0.01 M Tris–HCl, pH 6.3) and incubated overnight at 4°C with buffer E (8 M urea, 0.1 M NaH_2_PO_4_ and 0.01 M Tris–HCl, pH 4.5). Buffer exchange was performed using PD-10 desalting columns (Amersham Biosciences), replacing the acidic urea buffer with PBS (pH 7.2). Protein concentration was measured using a Nanodrop ND-1000 spectrophotometer (NanoDrop Technologies) by measuring the absorbance at 280 nm, and proteins were stored at −20°C.

### 5.9. Immunoblotting

Cells were lysed with RIPA buffer supplemented with Phosstop (Merck Millipore) and complete mini EDTA-free protease inhibitor cocktail. Alternatively, cells were fractionated using a cell fractionation kit (Thermo Fisher Scientific) as described in the manufacturer's instructions. Protein samples were separated on 4–20% gradient SDS–PAGE (Thermo Scientific) at 125 V and calibrated with Colourplus broad pre-stained marker. Gels were transferred to nitrocellulose membranes (BioRad) at 10 V for 30 min on a BioRad semi-dry transfer system. Membranes were blocked in Tris-buffered saline with 0.1% Tween20 (TBS-T) containing 5% bovine serum albumin (BSA, w/v). Primary antibodies (in TBS-T/5% BSA) were incubated with the membrane at either 4°C overnight or 1 h at room temperature. Membranes were washed (3 × 15 min) in TBS-T and subsequently probed with conjugated secondary antibody (in TBS-T/5% BSA) for 45 min at room temperature. The membrane was washed (5 × 10 min) with TBS-T before membranes were exposed to ECL substrate (Luminata Crescendo; Millipore) for visualization of immuno-reactive proteins. Antibodies used included anti-67LR (MLuC5), anti-37-kDa LAMR1 (A7 or IHLR) or anti-Galectin-3 (9H3.2).

### 5.10. Modelling the molecular interaction of galectin-3 with LAMR1

The structures of the Gal-3 CRD and LAMR1 were obtained from the RCSB protein data bank (www.rcsb.org). The molecular docking of Gal-3 (3zsj.pdb: lactose-liganded structure; 3zsm.pdb: non-liganded structure) and LAMR1 (3bch.pdb) was performed employing a server in zdock.umassmed.edu [60,61] based on current knowledge of crystal structures. RasMOL [62] and UCSF Chimera [63] were used to generate images of the molecular interactions and docking.

### 5.11. Bacterial strains, growth conditions and invasion assays

*Neisseria meningitidis* clinical isolates (electronic supplementary material, table S1) were grown on chocolate horse blood (Oxoid) at 37°C, in an atmosphere of 5% CO_2_. Mutagenesis of *N. meningitidis* MC58 *pilQ* and *porA* was described previously [7]. To mutate *pilE,* chromosomal DNA extracted from *N. meningitidis* C311Δ*pilE* (kindly provided by Prof. C. Tang, University of Oxford, UK) was used to mutate MC58 by natural transformation and allelic exchange as described previously [64]. The MC58Δ*lgtF* strain used in this study was described previously [65]. For selection of mutants, meningococcal cells were cultured on Mueller–Hinton agar plates supplemented with 1% Vitox (Oxoid) and, where appropriate, with streptomycin and spectinomycin (100 μg ml^−1^) or kanamycin (50 μg ml^−1^). Invasion assays were performed as previously described [66].

### 5.12. Expression and purification of recombinant LAMR1

The 37LRP coding sequence was generated with flanking *Nde*I and *Not*I restriction sites using the following primer sequences 5′-GGGAATTCCATATGGAGGTGCTATTCCAGGGACCCGGATCCATGTCCGGAGCCCTTGAT-3′ (forward primer) and 5′-AAGGAAAAAAGCGGCCGCTTAAGACCAGTCAGTGG TTGCT-3′ (reverse primer). The amplified PCR product was *Nde*I/*Not*I digested and ligated into ss-Fc-IRES-Tpz-pEFBOS [67], allowing the expression of N-terminally Fc-tagged 37LRP. Mutations were introduced by site-directed mutagenesis (QuikChange, Stratagene) using the following primer sequences: for 37LRP^R155A^, 5′-TAACACAGATTCTCCTCTGGCCTATGTGGACATTGCCATC-3′ (forward primer) and 5′-GATGGCAATGTCCACATAGGCCAGAGGAGAATCTGTGTTA-3′ (reverse primer); for 37LRP^K166A^, 5′-CCATCCCATGCAACAACGCGGGAGCTCACTCAGTGG-3′ (forward primer) and 5′-CCACTGAGTGAGCTCCCGCGTTGTTGCATGGATGG-3′ (reverse primer); and for 37LRP^Y139F^, 5′-CCTCTCACGGAGGCATCTTTTGTTAACCTACCTA-3′ (forward primer) and 5′-TAGGTAGGTTAACAAAAGATGCCTCCGTGAGAGG-3′ (reverse primer). Recombinant 37LRP was expressed in human embryonic kidney (HEK293T) cells grown in DMEM (Gibco) supplemented with 1% (v/v) antibiotic/antimycotic solution, 10% (v/v) FBS and 0.5% l-glutamine (Sigma). Transfection was achieved using CaPO_4_ precipitation. Briefly, DNA/CaCl_2_ mix was added to an equal volume of 2 × HEPES-buffered saline (pH 7.12), incubated for 10 min at room temperature, and then added to approximately 40% confluent HEK293T monolayers. After overnight incubation, the medium was replaced with Ultra CHO cell medium (Gibco) and cells incubated for a further 48 h. Cells were lysed with RIPA buffer (supplemented with Phosstop (Merck Millipore) and complete Mini EDTA-free protease inhibitor cocktail) and cell debris removed by centrifugation at 20 000*g* for 10 min at 4°C. Recombinant 37LRP was then purified by protein A affinity chromatography using HiTrap protein A–sepharose HP columns and the AKTA PrimePlus purification system, according to the manufacturer's instructions (GE Healthcare, Amersham, UK). Briefly, the clarified lysate was mixed with an equal volume of binding buffer, containing 20 mM sodium phosphate and 150 mM NaCl (pH 7.3), and then applied to the column. After washing unbound proteins, using the same buffer, 37LRP was eluted using 0.1 M glycine (pH 2.5), and the pH neutralized with 1 M Tris–HCl (pH 8.8). Eluted proteins were then dialysed into PBS.

### 5.13. Enzyme-linked immunosorbant assays

100 μl lactose-purified recombinant human Gal-3 (Calbiochem) or BSA (5 µg ml^−1^) in PBS was used to coat amino-reactive 96-well microtiter plates (Immobilizer Amino; Nunc) overnight at 4°C. Bacterial strains were grown in liquid culture, washed and labelled with digoxigenin (Roche) as described previously [51]. Labelled bacteria were added to ELISA plates for 2–4 h at room temperature. Plates were washed with PBS/T and incubated with 100 μl polyclonal anti-digoxigenin F_ab_ fragment–alkaline phosphatase antibody (1 : 5000; Roche) in PBS/1% BSA for 1 h, and then washed several times as described above. 100 µl of alkaline phosphatase substrate (5 mg ml^−1^; Roche) was added to each well, and the absorbance measured at 405 nm after 15 min using an ELISA reader (Biotek EL800). Inhibition assays were performed as described above, except that bacteria were pre-incubated with lactose or sugars for 2 h at room temperature before being added. For LamR/Gal-3 binding, 100 μl aliquots of 8.7 µg ml^−1^ 37LRP proteins were immobilized as above. Following washing in PBS/T, wells were blocked with 1% BSA/PBS for 1 h. 100 μl of 5 µg ml^−1^ Gal-3 was then added and incubated at room temperature for 1 h. After washing, 100 μl of mouse anti-Gal-3 (9H3.2; 1 : 8000 diluted in 1% BSA/PBS) was added and incubated at 4°C overnight. After washing, 100 μl anti-mouse IgG-HRP conjugate (1 : 8000 diluted in 1% BSA/PBS) was added and incubated at 4°C overnight. Plates were again vigorously washed and colour developed by adding 100 μl ABTS substrate (Roche). Plates were read at an absorbance of 405 nm.

### 5.14. Cross-linking

Cross-linking was performed as described previously [41]*.* Briefly, *N. meningitidis* was incubated with Gal-3 conjugated to the light-activated cross-linker Sulfo-SBED. After photoactivation, in which the reactive biotin moiety is transferred to molecules in close proximity to the cross-linking agent, cells were washed, lysed, subjected to SDS–PAGE and immunoblotting, and probed with streptavidin before molecules were identified using MALDI-TOF.

## Supplementary Material

Electronic supplementary material
