# Supplementary material for: Deciphering the complex three-way interaction between the non-integrin laminin receptor, galectin-3 and Neisseria meningitidis
Source: Open Biol. 2014 Oct 1;4(10):140053. doi: 10.1098/rsob.140053 (PMC4221890; doi:10.1098/rsob.140053)
Supplement: Electronic supplementary material [file rsob140053supp1.doc]

**Deciphering the complex three-way interaction between the non-integrin laminin receptor, galectin-3 and *Neisseria meningitidis***

**Fulwah Alqahtani^1†^, Jafar Mahdavi^1†*^, Lee M. Wheldon^1^, Necmettin Pirinccioglu^2^ Matthew Vassey^1^, Pierre-Joseph Royer^1^, Sozan M. Qarani^1^, Shaun Morroll^1^, Jeroen Stoof^1^, Nicholas D. Holliday^1^, Neil J. Oldfield^1^, Karl G. Wooldridge^1*^ and Dlawer A.A. Ala’Aldeen^1^**

^1^ School of Life Sciences, University of Nottingham, Nottingham, NG7 2RD, UK

^2^ Department of Chemistry, University of Dicle, TR-21280, Diyarbakir, Turkey

^†^ These authors contributed equally to this work

* For correspondence. Mailing address: Centre for Biomolecular Sciences, University of Nottingham, University Park, Nottingham, NG7 2RD, United Kingdom. Phone: +44 (0)115-823-0738 (JM) or +44 (0)115-823-0743 (KGW). Fax: +44 (0)115-846-8002. E-mail: [jafar.mahdavi@nottingham.ac.uk](mailto:jafar.mahdavi@nottingham.ac.uk) or karl.wooldridge@nottingham.ac.uk.

**SFigure 1:** **37LRP and 67LR can be differentiated using distinct antibodies.** Non-permeabilized hBMECs were stained with: (A) the commercially available mouse monoclonal anti-LamR1 (A7), (B) a rabbit polyclonal anti-LamR1 (IHLR), raised against very similar LamR1-derived peptides (aa253-289 and aa263-282, respectively). (C) A mouse monoclonal that has a reported specific immuno-reactivity for 67LR (MLuC5) and exhibits a different punctate staining pattern. All three antibodies were used to probe immunoblots of fractionated whole cell lysates: C, cytoplasmic; M, membrane; SN, soluble nuclear; CN, chromatin-associated nuclear; Cy, cytoskeletal. (D) Cell surface co-staining with both 37LRP-specific antibodies (A7 and IHLR) confirmed the similarity in staining pattern and a high degree of co-localization (81.5%, n=13 cells) illustrating similar cell surface targets. Scale bar = 10mm. Images are representative of at ≥3 independent experiments.

**
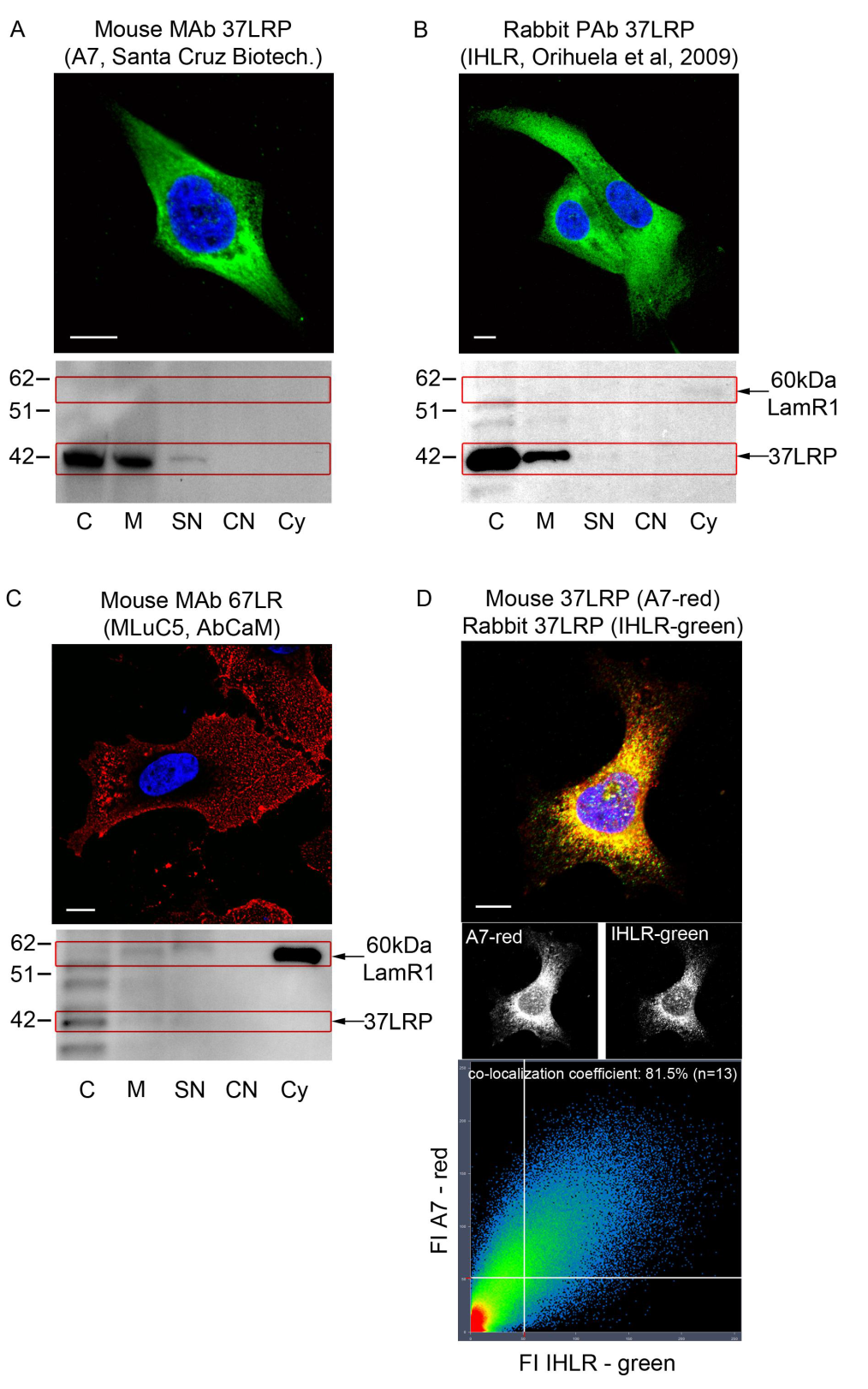
**

**SFigure 2:** **Expression of 37LRP in COS7 cells.** (A) Non-permeabilized COS7 cells were stained for endogenous 37LRP (green) and 67LR (red). Following transfection with either: (B) a 37LRP-GFP fusion, (C) a 37LRP-HA tagged construct or (D) both 37LRP-GFP and 37LRP-HA, cells were subsequently surface stained for 67LR (MLuC5). Despite some co-localization (yellow-arrowheads) of 37LRP-GFP and 37LRP-HA in (D), 67LR staining was consistently comparable to background staining from the anti-mouse IgM used to detect mLuC5. (E) Merged images in A-C include Hoechst 33258 DNA staining. Scale bar = 10mm.


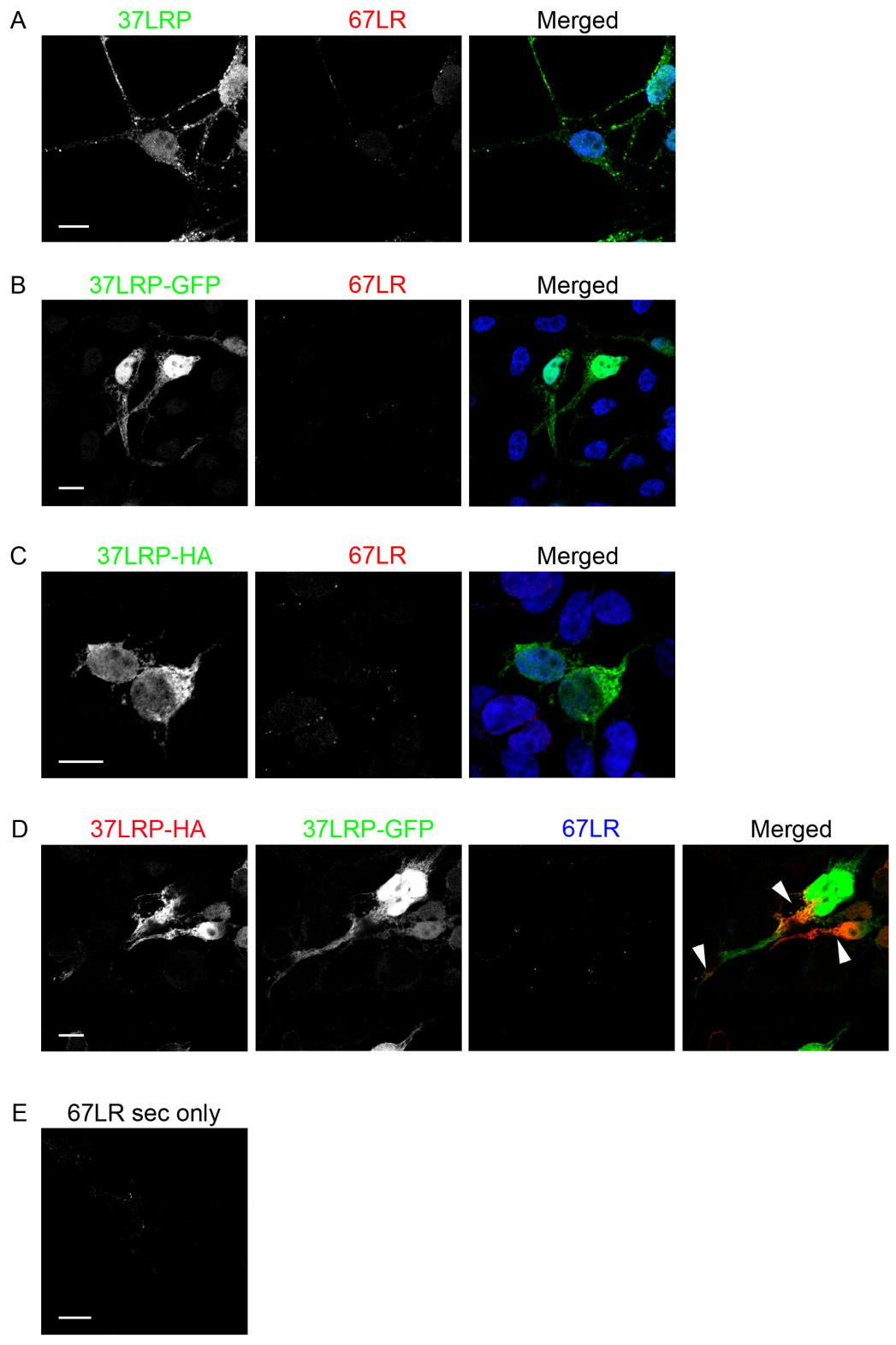


**SFigure 3. Expression of fluorescently labeled 37LRP and Gal-3 in transiently transfected COS7 cells.** COS7 cells were transiently transfected with constructs expressing either 37LRP (A) or Gal-3 (B). Lane 1: non-transfected cells (negative control). Lanes 2-4: target proteins fused to full length YFP or its N- (Yn-) or C- (Yc-) terminal domains, respectively. Proteins were detected on immunoblots using anti-GFP antibody.

**SFigure 4. Substitution of cysteines 148 and 163 of 37LRP do not affect 37LRP cell surface expression.** For flow cytometry analysis, Cos7 cells were used due to the aggregation of hBMECs. COS7 cells were transfected with BiFC constructs expressing alanine-substituted 37LRP and fluorescence levels were quantified. However, substitution of cysteines 148 and 163 of 37LRP do not affect 37LRP cell surface expression. Bars represent the mean ± s.e.m of three independent experiments. Bars represent the mean ± s.e.m. of three independent experiments.

**SFigure 5: Binding of a range of isolates of *N. meningitidis* to Lac-Gal-3.** *N. meningitidis* MC58 and 25 clinical isolates representing different clonal lineages were DIG-labeled and allowed to interact with Lac-Gal-3 in ELISA experiments. The data represent the mean OD at 405 nm ± s.e.m. (error bars) of samples tested in triplicate. Experiments were repeated three times with consistent results.

Table S1: *N. meningitidis* isolates used in this study

| Strain | Country of isolation | Date of isolation | Disease | Serogroup |
| --- | --- | --- | --- | --- |
| MC58^a^ | UK | 1983 | Invasive | B |
| Z1503^b^ | China | 1984 | Invasive | A |
| Z1035 ^b^ | Pakistan | 1967 | Invasive | A |
| Z4662 ^b^ | Netherlands | 1967 | Invasive | B |
| Z4665 ^b^ | Netherlands | 1977 | Invasive | B |
| Z6413 ^b^ | South Africa | 1990 | Invasive | C |
| Z6414 ^b^ | New Zealand | 1994 | Invasive | C |
| Z6415 ^b^ | England | 1996 | Invasive | C |
| Z3515 ^b^ | Saudi | 1987 | Carrier | A |
| Z1392 ^b^ | Greece | 1968 | Carrier | A |
| Z4686 ^b^ | Norway | 1988 | Carrier | B |
| Z4685 ^b^ | Norway | 1988 | Carrier | B |
| Z23279^c^ | UK | 2009 | Carrier | B |
| Z23288 ^c^ | UK | 2009 | Carrier | B |
| Z23000 ^c^ | UK | 2009 | Carrier | B |
| Z23464 ^c^ | UK | 2009 | Carrier | B |
| Z22955 ^c^ | UK | 2009 | Carrier | B |
| Z22951 ^c^ | UK | 2009 | Carrier | Y |
| Z22972 ^c^ | UK | 2009 | Carrier | Y |
| Z23484 ^c^ | UK | 2009 | Carrier | Y |
| Z99615 ^c^ | UK | 2010 | Carrier | Y |
| Z22984 ^c^ | UK | 2009 | Carrier | Y |
| Z1506 ^b^ | Brazil | 1976 | Unspecified | A |
| Z4262 ^b^ | USA | 1964 | Unspecified | B |
| Z4765 ^b^ | Brazil | 1976 | Unspecified | C |
| Z5163 ^b^ | Spain | 1985 | Unspecified | C |

^a^ Strain obtained from American Type Culture Collection (ATCC)

^b^ Strains obtained from Prof D. Caugant; more details are available at [**pubmlst.org**](http://pubmlst.org/)

^c^ Strains isolated in Nottingham, UK

**SFigure 6: Meningococcal binding to Lac-Gal-3 in the presence of potentially inhibitory sugars.** Binding of DIG-labeled *N. meningitidis* MC58 to immobilized Lac-Gal-3 was not inhibited by pre-incubation with different concentrations of lactose (A) or various sugars (all at 30 µg ml^-1^) (B). BSA-coated wells were included as a negative controls; the mean value obtained from the bacteria-BSA coated wells was subtracted from the Lac-Gal-3 coated wells. Binding of the MC58 in the absence of sugars served as a positive control. Data represent the mean OD at 405 nm ± s.e.m. (error bars) of a sample tested in triplicate. Experiments were repeated three times with consistent results.

**Materials and Methods**

***Cell culture***

All cells were cultured at 37°C, 5% CO_2_. Human brain microvascular endothelial cells were cultured in Endoprime media [PAA] supplemented with 5% (v/v) fetal bovine serum (FBS), IGF, bFGF, ascorbic acid, hydrocortisone and heparin, EGF, VEGF [all from PAA] and 1% antibiotic-antimycotic solution (v/v) [Gibco]. Cells were either P2-15 primary cells [Sciencell, USA] or immortalized cells at either early (P10-30) or late (P60-70) passage. Where cells were routinely cultured on fibronectin, fibronectin-coated T75 flasks were used [BD Biosciences]. Neuro 2a (N2a) mouse neuroblastoma cells were cultured in Eagle's Minimum Essential Medium (EMEM) supplemented with 1% (v/v) antibiotic-antimycotic solution and 10% (v/v) FBS. COS7 cells were grown in Dulbecco’s modified Eagle’s medium (DMEM, Invitrogen) supplemented with 1% (v/v) antibiotic-antimycotic solution (Gibco) and 10% (v/v) FBS. Cell culture media was changed every two days and cells were split using trypsin-EDTA (Gibco) upon reaching 90% confluence.

***Flow cytometry***

COS7 cells were grown in 6-well plates and detached 24 h after transfection using cell dissociation solution (Sigma), washed in PBA buffer (0.5% Bovine serum albumin [BSA], 0.5% sodium azide in PBS) and resuspended in 0.5% paraformaldehyde (PFA) in PBS (Oxoid) and subjected to flow cytometry using a Coulter Altra Flow Cytometer. Data acquisition and analysis were performed with Weasel 2.5 software. In each case 50,000 cells were counted in triplicate and used to calculate the average BiFC signal intensity ± SE. Full length YFP fluorescence signal was used as the inter-assay reference for maximal fluorescence in each experiment against which the BiFC signal intensities were compared.

***Immunofluorescence***

For confocal analysis, cells were grown on acid-etched glass 12mm coverslips [SLS] that were coated with 0.1% human fibronectin (1μl/2.5cm^2^ [Sigma]) or laminin10 (1μg/cm^2^ [Millipore]) as required. Following any treatments, cells were fixed with 4% PFA (10 min at RT) and, if required, permeabilized with 0.1% Triton X-100/1% BSA in PBS (5 min at RT). Following 1 h incubation in PBS/4% BSA to reduce non-specific binding, coverslips were incubated with primary antibody(s) either as a cocktail or sequentially, depending upon predetermined secondary antibody cross-talk: anti-67-kDa LamR1 (MAb MLuC5, 1:100, Abcam); anti-37-kDa LamR1 (PAb IHLR, 1:100, ; MAb A7, 1:250 [Santa Cruz Biotech.]; anti-Gal-3 (MAb M3/38, 1:50 [Biolegends]; MAb 9H3.2, 1:100 [Millipore]; PAb, 1:50 [R&D Systems]) in PBS-T/4% BSA for 1h. Coverslips were washed 3 times in PBS and incubated with conjugated secondary antibody(s): anti-mouse IgM (Alexa647, 1:1500; Alexa488, 1:1000); anti-mouse IgG (Alexa680, 1:200); anti-rabbit (Alexa488, 1:400; Alexa680 1:200); anti-goat (Alexa680, 1:200) (1:200); anti-rat (Alexa647, 1:200) all from Molecular Probes, in PBS-T/4% BSA for 1h and washed 3 times in PBS, once in H_2_O. Coverslips were mounted with Prolong Gold anti-fade with/without DAPI [Invitrogen] and images obtained as sections, unless stated otherwise, by confocal microscope. For each experiment, unstained and secondary antibody alone samples were processed in parallel to control for non-specific staining. Additionally, all possible primary/secondary combinations were checked for cross-reactivity and sequential staining used as required. For all co-localization studies, samples stained for localization of each protein individually were processed in parallel and in all cases the fluorescence of adjacent channels was monitored for bleed through.

***Confocal microscopy***

Images (400-600nm optical sections) were acquired using a Zeiss LSM 700 AxioObserver confocal microscope using a Plan-Apochromat 63x/1.40 Oil DIC M27 objective with Zen 2009 operating software. Images were processed using ImageJ and Adobe Photoshop software. Raw data are available upon request. Co‑localization analyses utilized the ZEN software function with a FI threshold of 50; the data was not intensity-weighted. Mean FI data of individual cells or fields of cells was also obtained using ZEN software.

***Bimolecular Fluorescence Complementation analysis***

BiFC expression constructs were obtained by PCR: cDNA sequences of human LamR1 (888bp) and Galectin-3 (725bp) were generated with flanking N-terminal EcoRI/Kozak and C-terminal XhoI restriction sites using the following primer sequences for Galectin-3: 5’‑GCGCGAATTCGCCATGGCAGACAATTTTTCGCT-3’ (forward primer) and 5’‑GCGCCTCGAGTATCATGGTATATGAAGCACTGGTG-3’ (reverse primer), and for LamR1: 5’-GCGCGAATTCGCCATGTCCGGAGCCCTTGAT-3’ (forward primer) and 5’‑GCGCCTCGAGAGACCAGTCAGTGGTTGCTCC-3’ (reverse primer). The amplified PCR products were digested and ligated into an EcoRI/XhoI-digested pcDNA3.1zeo vector, which contained either full length Venus Yellow Fluorescent Protein (vYFP) or the N- or C-terminal regions (Yn or Yc, respectively) of vYFP or full length mCherry. Cysteine mutations were introduced by site-directed mutagenesis [QuikChange, Stratagene] using the following primer sequences for Gal-3^C173A^: 5’‑GAGAACAACAGGAGAGTCATTGTTGCCAATACAAAGCTGGATAATAACTG-3’ (forward primer) and 5’‑CAGTTATTATCCAGCTTTGTATTGGCAACAATGACTCTCCTGTTGTTCTC-3’ (reverse primer), 37LRP^C148A^: 5’‑CCTACCTACCATTGCGCTGGCTAACACAGATTCTCCTCTG-3’ (forward primer) and 5’-CAGAGGAGAATCTGTGTTAGCCAGCGCAATGGTAGGTAGG-3’ (reverse primer) and 37LRP^C163A^: 5’-TGGACATTGCCATCCCAGCCAACAACAAGGGAGCTC-3’ (forward primer) and 5’-GAGCTCCCTTGTTGTTGGCTGGGATGGCAATGTCCA-3’ (reverse primer). Cells were grown on fibronectin-coated 12mm glass coverslips and transfected at approximately 60% confluence using TransIT-2020 at a DNA: TransIT-2020 ratio of 2:1 according to manufacturer’s instructions. In brief, preformed DNA:TransIT complexes were added to the growth media for 4 h. Cells were then washed with growth media and recombinant proteins expressed for 24 h before fixing with 4% PFA and subsequent confocal analysis.

***siRNA of Galectin-3 and LamR1***

hBMECs were cultured as described and transfected with predesigned siGENOME SMARTpool (mix of 4 siRNAs targeting one ORF) targeting LamR1 (M-013303-01-0005), Galectin-3 (M-010606-02-0005) or ON-TARGETplus non-targeting pool control siRNA [Dharmacon, Thermo Scientific]. Cells were transfected on day 1 with a final concentration of 37.5nM siRNA using the DharmaFECT transfection reagents [Dharmacon, USA]. The inhibition of LamR1 and Gal-3 expression was assessed on day 3 by confocal analysis, quantitative polymerase chain reaction (RT-qPCR) and immunoblot analysis. To determine transfection efficiency, non-transfected and siGLO-transfected cells were analyzed 1 day after transfection on a FACSCaliber machine [Beckton Coulter]. All Galectin-3 or LamR1 confocal images were acquired at the same resolution and scale, with the same hardware/laser settings used to image untreated control cells. Identical image manipulations were performed on all images stained with the same antibody set.

***qPCR analysis***

hBMECs were washed twice with serum-free EndoPrime base media [PAA] and total RNA extracted using the RNeasy Mini Kit [Qiagen] according to the manufacturer’s instructions. DNA was removed using RNAase-free Turbo-DNAase I [Ambion, Applied Biosystem]. RNA was cleaned and concentrated using RNeasy MiniElute Cleanup kit [Qiagen]. cDNA was synthesized using High Capacity cDNA Reverse transcription kit [Applied Biosystems]. RT-qPCR was performed in an ABI7500 Real time PCR system [Applied Biosystems] with the Brilliant SYBR Green qPCR Master Mix [Stratagene]. Cycling was initiated at 95°C for 10 min, followed by 40 cycles of 95°C for 15 s, 60°C for 60 s, and 60°C for 1 min. Samples were run in triplicate and relative expression of LamR1 and Galectin-3 was calculated using the comparative threshold cycle method normalized to GAPDH. Primers were designed using Primer 3 software and obtained from Sigma-Aldrich. Primer sequences were as follows: GAPDH (Forward: 5’-GGGAAACTGTGGCGTGAT-3′ and Reverse: 5’-TTCAGCTCAGGGATGACCTT-3’); LamR1 (Forward: 5’-CCATTGAAAACCCTGCTGAT-3’ and Reverse: 5’-CAGCGCAATGGTAGGTAGGT-3’); Galectin-3 (Forward: 5’-CTATAGCCGGGACTCCTTCC-3’ and Reverse: 5’-AGTTCCAGGGCACATACGTC-3’).

***Purification of recombinant protein***

Cells from 50 ml culture were re-suspended in 5ml of buffer B (8M urea [Sigma], 0.1M NaH₂PO₄ [BDH] & 0.01M Tris.HCl [Sigma] pH 8.0) and then sonicated in an ice bath for 15 cycles of 10 sec with 5 sec of cooling between cycles. Lysate was centrifuged at 10 000 *g* for 30-40 min at 4⁰C to pellet cellular debris, followed by incubation of the supernatant with 20mM imidazole (Qiagen) and cobalt resin (Fisher Scientific) overnight at 4˚C. Supernatant was passed through a gravity column and extensively washed with buffer C (8M urea, 0.1M NaH₂PO₄ & 0.01M Tris.HCl, pH 6.3) and incubated overnight at 4°C with buffer E (8M urea, 0.1M NaH₂PO₄ & 0.01M Tris.HCl, pH 4.5). Buffer exchange was performed using PD-10 desalting columns (Amersham Biosciences), replacing the acidic urea buffer with PBS (pH 7.2). Protein concentration was measured using a Nanodrop ND-1000 spectrophotometer (NanoDrop Technologies) by measuring the absorbance at 280 nm and proteins were stored at – 20°C.

***Immunoblotting***

Cells were lysed with RIPA buffer (supplemented with Phosstop [Merck Millipore] and c0mplete Mini EDTA-free protease inhibitor cocktail). Alternatively, cells were fractionated using a Cell Fractionation kit [Thermo Fisher Scientific] as described in the manufacturer’s instructions. Protein samples were separated on 4-20% gradient SDS-PAGE [Thermo Scientific] at 125V and calibrated with Colourplus broad pre-stained marker. Gels were transferred to nitrocellulose membranes [BioRad] at 10V for 30 min on a BioRad semi-dry transfer system. Membranes were blocked in TBS-T (Tris-buffered saline with 0.1% Tween20) containing 5% bovine serum albumin (BSA, w/v). Primary antibodies (in TBS-T/5% BSA) were incubated with the membrane at either 4^o^C overnight or 1 h at room temperature. Membranes were washed (3 x 15 min) in TBS-T and subsequently probed with conjugated secondary antibody (in TBS-T/5% BSA) for 45 min at room temperature. The membrane was washed (5 x 10 min) with TBS-T, before membranes were exposed to ECL substrate (Luminata Crescendo [Millipore]) for visualization of immuno-reactive proteins. Antibodies used included anti-67LR (MLuC5), anti-37-kDa LamR1 (A7 or IHLR) or anti-Galectin-3 (9H3.2).

***Modeling the molecular interaction of Galectin-3 with LamR1***

The structures of the Gal-3 CRD (carbohydrate recognition domain) and LamR1 were obtained from the RCSB protein data bank ([www.rcsb.org](http://www.rcsb.org/)). The molecular docking of Gal-3 (3zsj.pdb: lactose-liganded structure; 3zsm.pdb: non-liganded structure) and LamR1 (3bch.pdb) was performed employing a server in zdock.umassmed.edu based on current knowledge of crystal structures. RasMOL and UCSF Chimera were used to generate images of the molecular interactions and docking.

***Bacterial strains, growth conditions and invasion assays***

*N. meningitidis* clinical isolates (Table S1) were grown on chocolate horse blood (Oxoid) at 37°C, in an atmosphere of 5% CO_2_. Mutagenesis of *N. meningitidis* MC58 *pilQ* and *porA* was described previously . To mutate *pilE,* chromosomal DNA extracted from *N. meningitidis* C311Δ*pilE* (kindly provided by Prof. C. Tang, University of Oxford, UK) was used to mutate MC58 by natural transformation and allelic exchange as described previously . The MC58Δ*lgtF* strain used in this study was described previously . For selection of mutants, meningococcal cells were cultured on Mueller-Hinton agar plates supplemented with 1% Vitox (Oxoid) and, where appropriate, with streptomycin and spectinomycin (100 μg ml^-1^) or kanamycin (50 μg ml^-1^). Invasion assays were performed as previously described .

***Expression and purification of recombinant LamR1***

The 37LRP coding sequence was generated with flanking NdeI and NotI restriction sites using the following primer sequences 5’-gggaattcCATATGgaggtgctattcca gggacccggatccATGTCCGGAGCCCTTGAT-3’ (forward primer) and 5’-AAGGAAAAAAGCGGCCGCTTAAGACCAGTCAGTGGTTGCT-3’ (reverse primer). The amplified PCR product was NdeI/NotI digested and ligated into ss-Fc-IRES-Tpz-pEFBOS , allowing the expression of N-terminally Fc-tagged 37LRP. Mutations were introduced by site-directed mutagenesis [QuikChange, Stratagene] using the following primer sequences for 37LRP^R155A^: 5’-taacacagattctcctctggcctatgtggacattgccatc-3’ (forward primer) and 5’-Gatggcaatgtccacataggccagaggagaatctgtgtta-3’ (reverse primer), 37LRP^K166A^: 5’-ccatcccatgcaacaacgcgggagctcactcagtgg-3’ (forward primer) and 5’-ccactgagtgagctcccgcgttgttgcatggatgg-3’ (reverse primer) and 37LRP^Y139F^: 5’-cctctcacggaggcatcttttgttaacctaccta-3’ (forward primer) and 5’-taggtaggttaacaaaagatgcctccgtgagagg-3’ (reverse primer). Recombinant 37LRP was expressed in human embryonic kidney (HEK293T) cells grown in Dulbecco’s modified Eagle’s Medium (DMEM) (Gibco) supplemented with 1% (v/v) antibiotic/antimycotic solution, 10% (v/v) FBS and 0.5% L-Glutamine (Sigma). Transfection was achieved using CaPO_4_ precipitation. Briefly, DNA/ CaCl_2_ mix was added to an equal volume of 2 × HEPES buffered saline (pH 7.12), incubated for 10 min at room temperature, and then added to *ca.* 40% confluent HEK293T monolayers. After overnight incubation, the media was replaced with Ultra CHO cell media (Gibco) and cells incubated for a further 48 h. Cells were lysed with RIPA buffer (supplemented with Phosstop [Merck Millipore] and c0mplete Mini EDTA-free protease inhibitor cocktail) and cell debris removed by centrifugation at 20 000 *g* for 10 min at 4°C. Recombinant 37LRP was then purified by Protein A affinity chromatography using HiTrap™ Protein A-sepharose HP columns and the AKTA PrimePlus purification system, according to the manufacturer’s instructions (GE Healthcare, Bucks, UK). Briefly, the clarified lysate was mixed with an equal volume of binding buffer, containing 20 mM sodium phosphate and 150 mM NaCl (pH 7.3), and then applied to the column. After washing unbound proteins, using the same buffer, 37LRP was eluted using 0.1M glycine (pH 2.5), and the pH neutralized with 1M Tris-HCl (pH 8.8). Eluted proteins were then dialyzed into PBS.

*Enzyme-linked immunosorbant assays*

100 μl lactose-purified recombinant human Gal-3 (Calbiochem) or BSA (5 µg ml^-1^) in PBS were used to coat amino-reactive 96-well microtiter plates (Immobilizer Amino; Nunc) overnight at 4°C. Bacterial strains were grown in liquid culture, washed and labeled with digoxigenin (Roche) as described previously . Labeled bacteria were added to ELISA plates for 2-4h at room temperature. Plates were washed with PBS/T and incubated with 100 μl polyclonal anti-digoxigenin F_ab_ fragment–alkaline phosphatase antibody (1:5,000; Roche) in PBS/1% BSA for 1h and then washed several times as described above. 100μl of alkaline phosphatase substrate (5 mg ml^-1^; Roche) was added to each well, and the absorbance measured at 405nm after 15 min using an ELISA reader (Biotek EL800). Inhibition assays were performed as described above, except that bacteria were pre-incubated with lactose or sugars for 2 h at room temperature before being added. For LamR/Gal-3 binding, 100 μl aliquots of 8.7 µg ml^-1^ 37LRP proteins were immobilized as above. Following washing in PBS/T, wells were blocked with 1% BSA/PBS for 1 h. 100 μl of 5 µg ml^-1^ Gal-3 was then added and incubated at room temperature for 1 h. After washing, 100 μl of mouse anti-Gal-3 (9H3.2; 1:8,000 diluted in 1% BSA/PBS), was added and incubated at 4°C overnight. After washing, 100 μl anti-mouse IgG-HRP conjugate (1:8,000 diluted in 1% BSA/PBS), was added and incubated at 4°C overnight. Plates were again vigorously washed and color developed by adding 100 μl ABTS substrate (Roche). Plates were read at an absorbance of 405 nm.

***Cross-linking***

Cross-linking was performed as described previously *.* Briefly, *N. meningitidis* was incubated with Gal-3 conjugated to the light-activated cross-linker Sulfo-SBED. After photo-activation, in which the reactive biotin moiety is transferred to molecules in close proximity to the cross-linking agent, cells were washed, lysed, subject to SDS-PAGE and immunoblotting and probed with streptavidin before molecules were identified using MALDI-TOF.

**References:**

Abouseada, N.M., Assafi, M.S.A., Mahdavi, J., Oldfield, N.J., Wheldon, L.M., Wooldridge, K.G., and Ala’Aldeen, D.A.A. (2012). Mapping the laminin receptor binding domains of *Neisseria meningitidis* PorA and *Haemophilus influenzae* OmpP2. PLoS One *7*, e46233.

Akbarzadeh, S., Wheldon, L.M., Sweet, S.M.M., Talma, S., Mardakheh, F.K., and Heath, J.K. (2008). The deleted in Brachydactyly B Domain of ROR2 is required for receptor activation by recruitment of Src. PLoS ONE *3*, e1873.

Ala’Aldeen, D.A.A., Oldfield, N.J., Bidmos, F.A., Abouseada, N.M., Ahmed, N.W., Turner, D.P.J., Neal, K.R., and Bayliss, C.D. (2011). Carriage of meningococci by university students, United Kingdom. Emerg Infect Dis *17*, 1761-1763.

Hadi, H.A., Wooldridge, K.G., Robinson, K., and Ala'Aldeen, D.A. (2001). Identification and characterization of App: an immunogenic autotransporter protein of *Neisseria meningitidis*. Mol Microbiol *41*, 611-623.

Mahdavi, J., Royer, P.-J., Sjölinder, H.S., Azimi, S., Self, T., Stoof, J., Wheldon, L.M., Brännström, K., Wilson, R., Moreton, J.*, et al.* (2013). Pro-inflammatory cytokines can act as intracellular modulators of commensal bacterial virulence. Open Biology *3:130048*.

Mahdavi, J., Sonden, B., Hurtig, M., Olfat, F.O., Forsberg, L., Roche, N., Angstrom, J., Larsson, T., Teneberg, S., Karlsson, K.A.*, et al.* (2002). *Helicobacter pylori* SabA adhesin in persistent infection and chronic inflammation. Science *297*, 573-578.

Mintseris, J., Pierce, B., Wiehe, K., Anderson, R., Chen, R., and Weng, Z. (2007). Integrating statistical pair potentials into protein complex prediction. Proteins *69*, 511-520.

Oldfield, N.J., Bland, S.J., Taraktsoglou, M., Ramos, F.J.D., Robinson, K., Wooldridge, K.G., and Ala'Aldeen, D.A.A. (2007). T-cell stimulating protein A (TspA) of *Neisseria meningitidis* is required for optimal adhesion to human cells. Cell Microbiol *9*, 463-478.

Orihuela, C.J., Mahdavi, J., Thornton, J., Mann, B., Wooldridge, K.G., Abouseada, N., Oldfield, N.J., Self, T., Ala'Aldeen, D.A., and Tuomanen, E.I. (2009). Laminin receptor initiates bacterial contact with the blood brain barrier in experimental meningitis models. J Clin Invest *119*, 1638-1646.

Pettersen, E.F., Goddard, T.D., Huang, C.C., Couch, G.S., Greenblatt, D.M., Meng, E.C., and Ferrin, T.E. (2004). UCSF Chimera--a visualization system for exploratory research and analysis. J Comput Chem *25*, 1605-1612.

Pierce, B.G., Hourai, Y., and Weng, Z. (2011). Accelerating protein docking in ZDOCK using an advanced 3D convolution library. PLoS One *6*, e24657.

Sayle, R.A., and Milner-White, E.J. (1995). RASMOL: biomolecular graphics for all. Trends Biochem Sci *20*, 374.
